# Genotype‐dependent DNA methylation patterns are negatively associated with allelic variation rather than heat‐induced gene expression in two contrasting potato genotypes

**DOI:** 10.1111/tpj.70690

**Published:** 2026-01-18

**Authors:** Darren Sheng Gin Yeo, Julia Eydam, Julien Bruckmüller, Friedrich Kauder, Jens Lübeck, Alexander Kaier, Sophia Sonnewald, Uwe Sonnewald

**Affiliations:** ^1^ Department of Biology Friedrich‐Alexander‐Universität Erlangen‐Nürnberg Staudtstrasse 5 91058 Erlangen Germany; ^2^ Solana Research GmbH Eichenallee 9 24340 Windeby Germany; ^3^ Present address: NPZ Innovation GmbH Hohenlieth‐Hof 1 D‐24363 Holtsee Germany

**Keywords:** potato, heat stress, DNA methylation, allelic variation

## Abstract

Potato (*Solanum tuberosum* L.) is an important food crop that is sensitive to high temperatures, which cause major changes in the transcriptome and a reduction in yield. In several plant species, DNA methylation has been reported to influence gene expression, particularly under abiotic stress conditions. However, the role of DNA methylation in regulating gene expression in heat‐tolerant and heat‐sensitive potato genotypes is still poorly understood. In this study, we conducted genome‐wide DNA methylome and transcriptome analyses of leaves from two contrasting potato cultivars, Annabelle (moderately heat‐tolerant) and Camel (heat‐sensitive), before and after heat stress (HS). Genome‐wide differential methylation analysis revealed that most identified differentially methylated regions (DMRs) were constitutive, reflecting variation between cultivars rather than being induced by HS. While thousands of heat‐responsive differentially expressed genes (DEGs) were identified, only a small fraction coincided with heat‐induced DMRs. Despite substantial constitutive DNA methylation and transcriptome differences between the cultivars, we found no consistent association between DMRs and DEGs, indicating that DNA methylation does not play a widespread direct regulatory role in gene expression. Surprisingly, hypermethylated genomic regions were associated with lower alternative allele frequencies, whereas hypomethylated regions showed the opposite trend. These findings indicate that the potato DNA methylome is largely stable under HS and that constitutive DNA methylation variation contributes rather to genetic diversity than to the direct regulation of gene expression.

## Significance Statement

To determine the role of DNA methylation in heat stress (HS) adaptation, two contrasting potato cultivars, Annabelle and Camel, were subjected to severe HS conditions. Surprisingly, we found DNA methylation is relatively unaffected after HS in both cultivars and is associated with allelic variation.

## INTRODUCTION

Domesticated over 8000 years ago in the Andes, the potato (*Solanum tuberosum*) has since spread across the globe to become the world's third most consumed food crop after rice and wheat. Today, more than 5000 varieties, adapted to diverse climates, are cultivated in 159 countries, contributing to the diet of nearly two‐thirds of the global population. This diversity makes the potato a cornerstone of global food security and nutrition. In 2023, global production exceeded 380 million tons. Remarkably, despite a 17 percent decline in potato farmland between 2000 and 2020, total output rose by more than 11 percent, thanks to improved varieties and advances in agronomic practices (FAO, 2024). Despite its global importance, potato production faces growing challenges from climate change. Rising temperatures and shifting precipitation patterns create favorable conditions for the spread of insect vectors. A notable example is the reed glassy‐winged leafhopper, which transmits bacteria and phytoplasmas responsible for diseases such as syndrome basses richesses (Behrmann et al., 2023; Therhaag et al., 2024). These infections cause rubbery tubers, wilting leaves, and an increased proportion of small tubers, leading to severe yield losses and reduced quality. Warmer climates have facilitated the spread of this pest, which has also expanded its host range from sugar beet to potatoes and other vegetable crops (Behrmann et al., 2023; Therhaag et al., 2024).

Beyond vector‐borne diseases, elevated temperatures themselves pose a major threat to potato production. Potato yield is particularly sensitive to heat stress (HS), which is considered one of the most critical factors limiting growth and productivity (Levy & Veilleux, 2007). Daytime temperatures above 27°C markedly reduce tuber yields in many cultivars (Hancock et al., 2014; Hastilestari et al., 2018; Wolf et al., 1990). This effect is more pronounced under long‐day conditions, highlighting a strong interaction between photoperiod and temperature (Wolf et al., 1990). Exposure to high temperatures early in development can inhibit or even completely suppress tuber formation (Wolf et al., 1990). With global warming accelerating, the development of heat‐tolerant potato varieties is therefore an urgent priority (Levy & Veilleux, 2007).

Stress responses in plants are highly complex and regulated by multiple mechanisms that ultimately lead to the up‐ or downregulation of genes enhancing fitness under adverse conditions. Importantly, traits that are beneficial during stress may be disadvantageous under normal conditions (Bao et al., 2020; Cipollini et al., 2017; Herms & Mattson, 1992). Therefore, stress responses must remain transient, and the expression of stress‐responsive genes should be reversible. While permanent genome mutations cannot provide the necessary flexibility for transient adaptations, epigenetic regulatory mechanisms—such as DNA methylation and histone modifications, often mediated by non‐coding RNAs (ncRNAs)—offer a dynamic and reversible means of adjusting gene expression (Zhang et al., 2018). Nevertheless, genotype‐specific stress responses must also involve stable genetic differences, such as mutations or irreversible modifications, which define the inherent variation among cultivars. Thus, distinguishing stable genetic determinants from reversible epigenetic modifications is critical for understanding plant stress adaptation.

In plants, the link between stress responses and epigenetic regulation has been recognized for decades (Chinnusamy & Zhu, 2009; Choi & Sano, 2007; Grativol et al., 2012; Kovarik et al., 1997; van Dijk et al., 2010; Zhu et al., 2008). However, only with the advent of genome‐wide association studies, combined with transcriptomic and methylomic analyses, it became possible to unravel the complex polygenic basis of stress tolerance (Singh et al., 2019). For example, global run‐on sequencing (GRO‐seq) and transcriptome analyses have demonstrated that HS tolerance is a polygenic trait, involving the coordinated up‐ and down‐regulation of numerous genes (Li et al., 2019; Liu et al., 2021; Singh et al., 2019). Similarly, microarray studies in potato cultivars with contrasting tuberization responses under HS revealed thousands of differentially expressed genes (DEGs) (>2‐fold change) between tolerant and sensitive genotypes (Singh et al., 2015). Although identifying specific candidate genes directly involved in tuberization remains challenging, many of these DEGs belong to general stress‐ or HS‐responsive categories.

Among the key regulators, HEAT SHOCK TRANSCRIPTION FACTORS (HSFs) play a central role. Knockdown or knockout of HSF genes in tomato and Arabidopsis significantly reduces the induction of HS‐responsive genes, underscoring their importance. In potato, Trapero‐Mozos et al. (2018) reported that a specific *HSc70* allele conferred improved heat tolerance when introduced into a heat‐susceptible genotype, suggesting that the upregulation of heat shock proteins (HSPs) can enhance thermotolerance. In parallel, tuberization regulators such as *SP6A* and *BEL5* are well‐characterized (Banerjee et al., 2006; Lehretz et al., 2019), though their epigenetic regulation under HS remains largely unexplored and warrants further investigation.

A fundamental question in understanding HS tolerance and temperature‐dependent tuberization is how heat‐responsive and tuberization‐related genes are activated and repressed. Potential mechanisms include temperature‐induced changes in protein stability or structure, as well as sequence‐level variation such as single nucleotide polymorphisms (SNPs) in genes or regulatory elements that modulate sensitivity and tolerance (Maulana et al., 2018). Beyond these, epigenetic regulation has emerged as a significant contributor to stress responses, including HS tolerance (Liu et al., 2015; Naydenov et al., 2015). Through DNA and histone modifications, epigenetic mechanisms reshape chromatin structure and influence the accessibility of genomic regions for the transcriptional machinery (Henderson & Jacobsen, 2007). In particular, DNA methylation, primarily the addition of methyl groups to cytosines or their demethylation, plays a pivotal role. In plants, DNA methylation can occur in CG, CHG, and CHH sequence contexts and is maintained by separate mechanisms. CG methylation is maintained by the methyltransferase 1 (MET1), which recognizes hemi‐methylated CG sites after DNA replication and methylates the unmethylated cytosine in the daughter strand. CHG methylation is primarily maintained by CHROMOMETHYLASE 3 (CMT3), with CHROMOMETHYLASE 2 (CMT2) contributing to a lesser extent. CHH methylation is sustained either by CMT2 or DOMAINS REARRANGED METHYLASE 2 (DRM2) (Niederhuth et al., 2016; Zhang et al., 2018). All three sequence contexts are initially methylated *de novo* by the RNA‐directed DNA methylation (RdDM) pathway, in which double‐stranded RNAs (dsRNAs) target specific loci for cytosine methylation (Pélissier et al., 1999; Wassenegger et al., 1994).

Evidence from Arabidopsis shows that RdDM is required for basal heat tolerance, and HS can alter the expression of genes involved in DNA methylation, thereby reshaping the methylome (Boyko et al., 2010; Popova et al., 2013). This highlights the importance of integrating transcriptome (gene expression) data with methylome (DNA methylation) profiles to gain a comprehensive understanding of stress adaptation. In addition to stress adaptation, DNA methylation has also been shown to be associated with genetic variation in various plant species (Dubin et al., 2015; Eichten et al., 2013; Galanti et al., 2022; Kusmartsev et al., 2020; Sammarco et al., 2024).

In this study, we investigated the role of global DNA methylation in differentiating two potato genotypes with different levels of heat response. By comparing their epigenomes before and after HS conditions and correlating differential methylation patterns with transcriptome changes, we aimed to unravel the potential role of epigenetic regulation in potato HS adaptation.

## RESULTS

### Impact of HS on two contrasting potato cultivars

In this study, we aimed to determine whether constitutive or inducible changes in genome‐wide DNA methylation patterns contribute to the differential responses of two contrasting potato varieties to HS. The varieties selected were Annabelle (AA) and Camel (CA). Annabelle is an early tuber‐forming, moderately heat‐tolerant cultivar, whereas Camel is a mid‐early tuber‐forming cultivar that is more heat‐sensitive (Yeo et al., 2025).

As described in Materials and Methods, plants were grown for 28 days under control conditions (21°C/19°C) before being subjected to HS (37°C/35°C) for 3, 7, 14, 21, or 28 days. After exposure, all plants were allowed to recover under control conditions for 14 days (Figure S1). Our aim was to select the strongest stress condition at which the potato plants still survive. We found that plants exposed to HS for 21 days were at the threshold of recovery (Figure S2). Plants grown for 14 days under HS recovered fully (Figure S3A), whereas those exposed for 28 days showed incomplete recovery (Figure S3B). While tuber number was unaffected (Camel) or slightly increased (Annabelle) (Figure S4A), tuber yield decreased progressively with longer durations of HS in both cultivars, but the reduction was more pronounced in the heat‐sensitive Camel cultivar as expected (Figure S4B).

### Genome‐wide DNA methylation profile under HS

DNA methylation has often been associated with plant adaptations to stressful conditions (Dubin et al., 2015; Thiebaut et al., 2019). In potato, HS has been shown to alter the expression of cytosine‐5 DNA methyltransferases (*C5‐MTases*) and demethylases (*DeMets*) in a genotype‐specific manner (Dutta et al., 2022). This led the authors to speculate that high temperature stress regulates the expression of tuberization factors via epigenetic modifications. To test whether DNA methylation patterns differ between heat‐sensitive and heat‐tolerant genotypes and whether HS modulates DNA methylation patterns, a methylome study was conducted. Leaf samples, prior to and 21 days after the application of HS, were analyzed using whole genome bisulfite sequencing (WGBS), enabling the identification of constitutive and heat‐induced DMRs.

**Figure 1:**
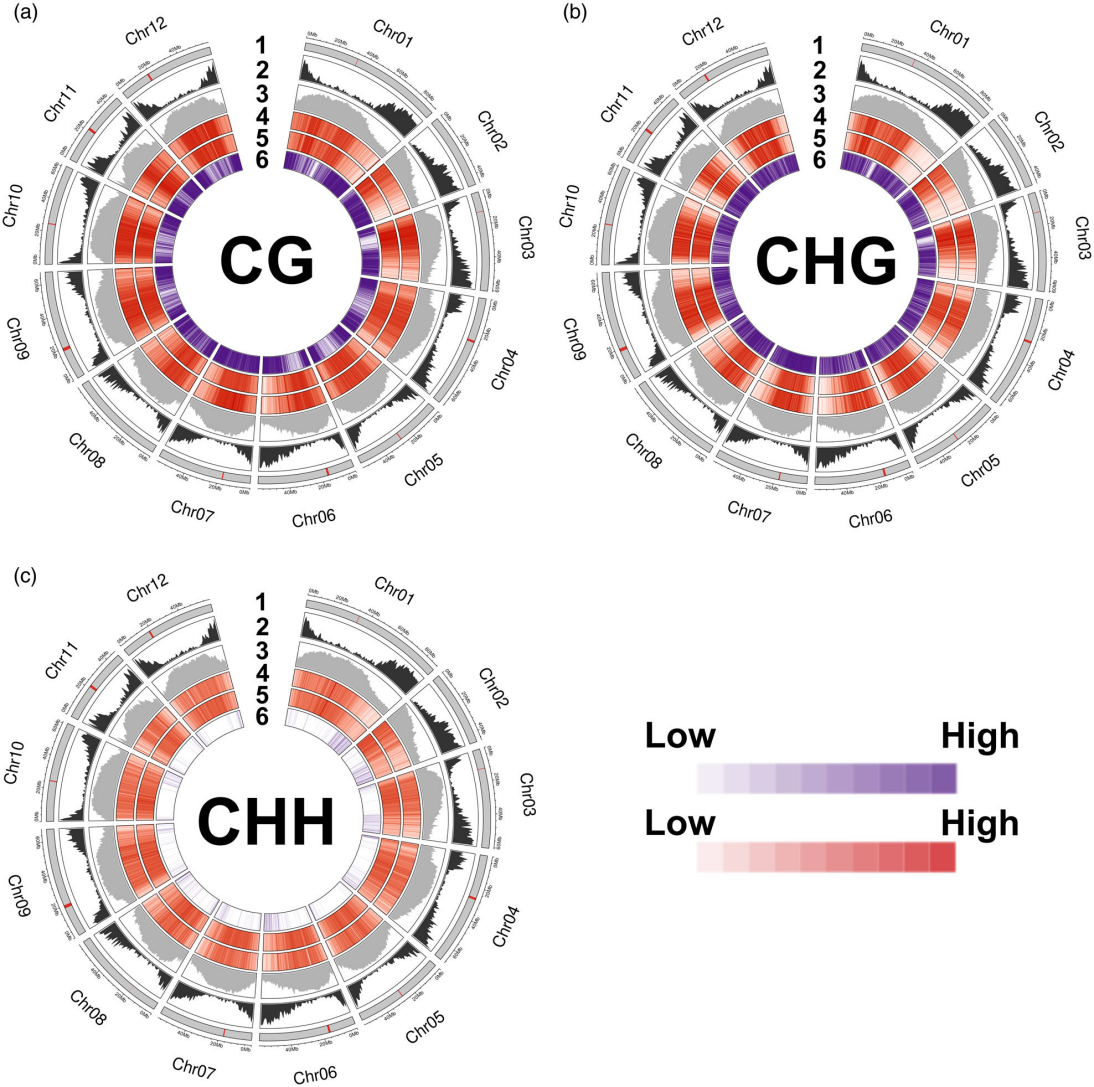
Circular representation of genomic feature distribution. (1) Chromosome ideogram of the v6.1 *Solanum phureja* reference genome. The centromeres are indicated by red bars, with the thickness corresponding to the relative size of the centromeric region on each chromosome (table S5 of Pham et al. (2020)). (2) Gene density and (3) repetitive sequences density frequency plot according to v6.1 *S. phureja* reference genome. DNA methylation density of (4) Annabelle and (5) Camel in CG (a), CHG (b) and CHH (c) context calculated genome‐wide within 500 kb windows. Genomic positions of identified constitutive DMRs between Annabelle and Camel (6) in (a) CG, (b) CHG and (c) CHH context.

The constitutive genome‐wide DNA methylation density in the two potato cultivars was determined in the leaf samples before HS at the chromosome level using a 500 kb sliding window (Figure 1a–c). In accordance with the v6.1 *Solanum phureja* reference genome, pericentromeric regions in both cultivars are rich in repetitive elements, whereas gene‐dense regions tend to contain fewer repeats. We found that DNA methylation density in the CG, CHG, and CHH contexts is highest in these repetitive pericentromeric regions consistent with findings in other plant species (An et al., 2017; Seymour et al., 2014; Shi et al., 2021). Gene‐rich regions typically showed a lower degree of DNA methylation across all three contexts. Interestingly, we found that variation in DNA methylation between Annabelle and Camel is most pronounced in gene‐rich regions, particularly in the CG and CHH contexts (Figure 1a,c). These results suggest that, as in other plants, DNA methylation in potatoes likely serves as an evolutionarily conserved mechanism to repress transposable element (TE) activity (He et al., 2022; Niederhuth et al., 2016; Rabinowicz et al., 1999).

To assess whether overall DNA methylation is affected by HS, we analyzed average methylation levels in our samples before and after heat treatment. Therefore, the reference genome was divided into 200 bp bins, and a weighted average methylation level was calculated for each window in the CG, CHG, and CHH contexts (Schultz et al., 2012).

**Figure 2:**
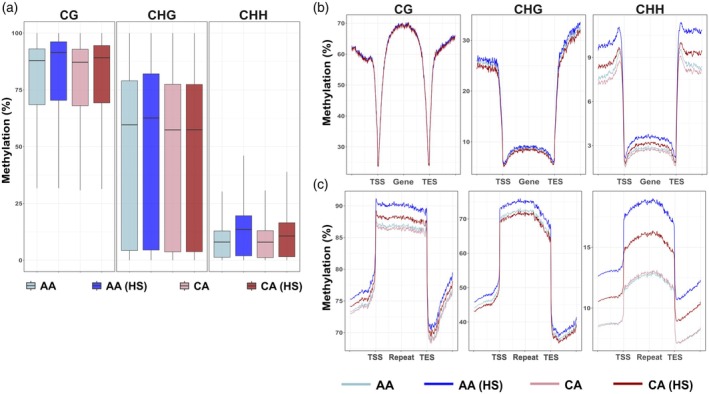
Genome‐wide global methylation distribution. (a) Global methylation distribution of all methylation context in 200 bp windows across the reference genome. (b) Metagene plot of all methylation context within 2 kb upstream of transcription start site (TSS), gene body and within 2 kb downstream of transcription end site (TES). (c) Metagene plot of all methylation context within 2 kb upstream and 2 kb downstream of repetitive elements.

Using these 200 bp windows, we observed that CG and CHG methylation levels followed a bimodal distribution, while CHH methylation was strongly skewed toward zero (indicating very low methylation) in all samples (Figure S5), CG methylation was the highest, with values typically ranging from 60 to 95%. CHG methylation was more variable, spanning from 2 to 80% across the genome. In contrast, CHH methylation was sparse, with levels between 1 and 17% (Figure 2a; Figure S5).

When comparing methylation levels before and after HS in Annabelle and Camel, we observed a slight overall increase in methylation across all contexts post stress, except for CHG methylation in Camel, which showed no notable increase.

Next, we analyzed methylation patterns around gene features, using a 2 kb window upstream and downstream of the gene bodies (Figure 2b). This revealed a high level of CG methylation within gene bodies consistent with the presence of gene body methylation (gbM), which predominantly occurs in the CG context (Niederhuth et al., 2016). In contrast, CHG and CHH methylation remained low within gene bodies. A typical methylation pattern was observed: hypermethylation in the upstream and downstream flanking regions, and hypomethylation near the transcription start site (TSS) and transcription end site (TES) (Niederhuth et al., 2016). Methylation patterns in gene regions were largely similar between samples, except for a slight increase in CHH methylation in flanking regions after HS.

We also assessed DNA methylation around repetitive elements, again using 2 kb windows upstream and downstream. These regions were generally highly methylated across all contexts. After HS, repetitive elements exhibited increased methylation, particularly in the CHH context (Figure 2c). This suggests that HS induces CHH hypermethylation in repetitive elements, likely as a protective mechanism to suppress overall repetitive element activity. To explore this further, we examined TE expression based on repetitive elements annotated in the v6.1 *S. phureja* reference genome. In general, we observed an overall increase in TE expression in both cultivars following HS (Figure S6). A closer inspection revealed that over 500 putative TEs were upregulated and fewer than 100 were downregulated after HS in both Annabelle and Camel (Table S1). This indicates that, despite the observed hypermethylation, HS also triggered increased TE expression in both cultivars. The results suggest that heat‐induced CHH hypermethylation may serve to counterbalance TE activation under stress conditions.

### Identification of constitutive and HS associated DNA methylation variation

To get insights into genotype‐specific DNA methylations, heat‐induced and constitutive DMRs between Annabelle and Camel were determined. Induced DMRs were identified by comparing samples before (28 dap) and after (49 dap) HS, while constitutive DMRs were identified between Annabelle and Camel before (28 dap) HS. Based on the DMR identification, a higher number of constitutive DMRs were identified in comparison to heat‐induced DMRs (Table 1). Constitutive DMRs additionally appear to be concentrated in regions where the gene density is high in the CG and CHH context for most of the chromosomes (Figure 1). Interestingly, DMRs in the CHH context were the most abundant among all heat‐induced DMRs. This is consistent with previous studies showing that CHH methylation is more likely to be induced when grown in variable environmental conditions (Dubin et al., 2015; Secco et al., 2015). The heat‐tolerant cultivar Annabelle had more induced DMRs in comparison to the heat‐sensitive Camel cultivar for all methylation contexts. This indicates that most of the DNA methylation variations are genotype‐dependent and are not induced by HS.

**Table 1:** Summary of DMR counts in different methylation sequence contexts

DMR type	Comparison	Methylation sequence context					
		CG		CHG		CHH	
		Hyper	Hypo	Hyper	Hypo	Hyper	Hypo
Constitutive	AA (compare group) versus CA	17 228	18 409	18 189	11 832	496	277
Heat inducible	AA (after) versus AA (before)	0	14	0	54	244	76
Heat inducible	CA (after) versus CA (before)	0	2	0	4	4	1

### Identifying heat‐induced DMR‐associated DEGs

**Figure 3:**
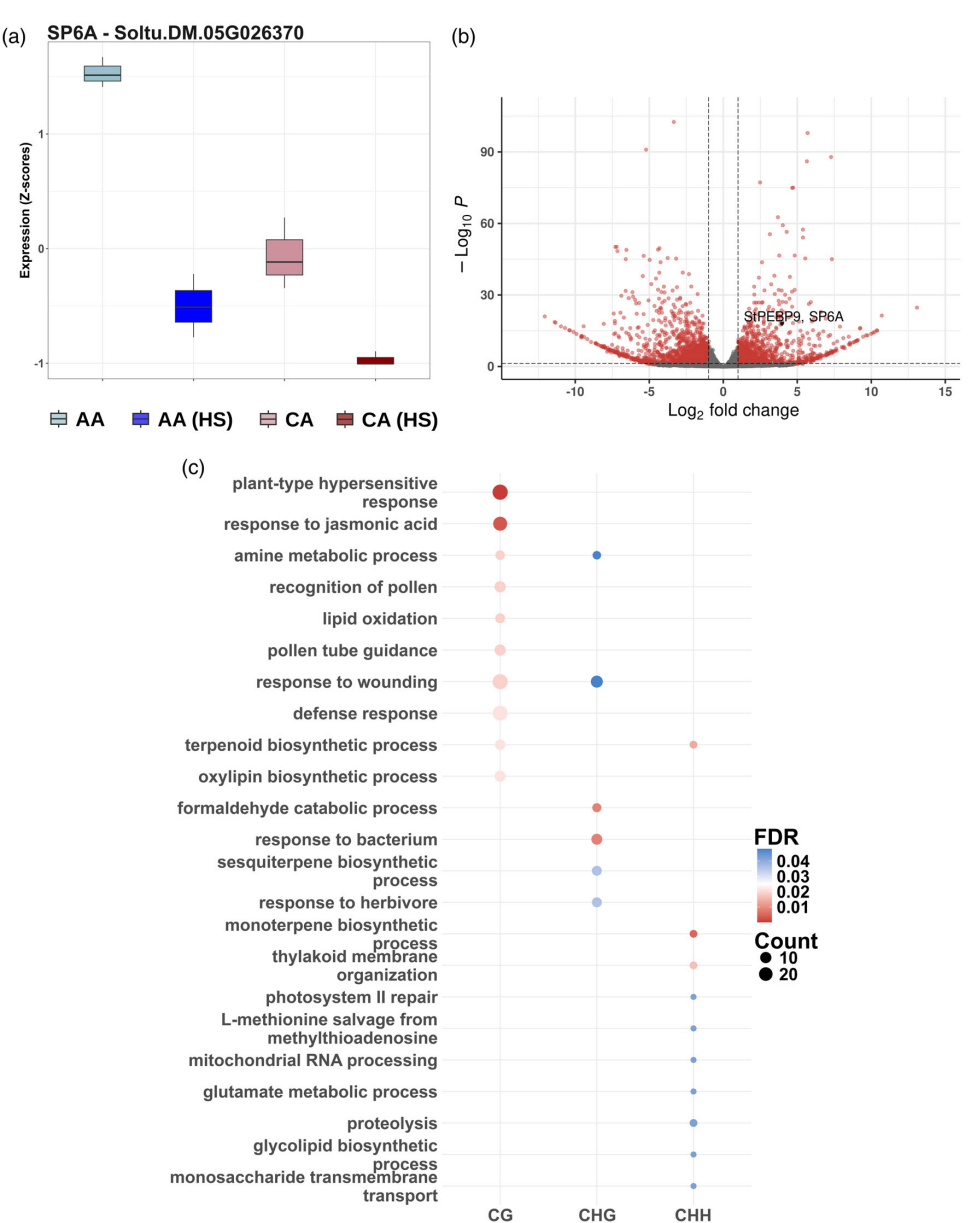
Constitutive DMRs and differentially expressed genes (DEGs) between Annabelle and Camel. (a) Difference in expression of *SP6A* between Annabelle (AA) and Camel (CA) represented by *z*‐scores on the *Y*‐axis. (b) Volcano plot of all identified DEGs between Annabelle and Camel. Genes were considered DEGs if FDR <0.05 and Log2 fold change >1 or <−1. Significant DEGs were represented in red dots while gray dots represent non‐significant DEGs. (c) Top 10 GO terms of GO enrichment by overrepresentation analysis for each gene body DMR DEGs separated into different methylation contexts.

Differential gene expression analysis was conducted to identify heat‐induced DEGs. A total of 4328 and 4087 upregulated genes were identified in the cultivars Annabelle and Camel, respectively, while 3576 and 3455 downregulated genes were detected in the same cultivars. Notably, the key tuberigen gene *SP6A* was downregulated in response to HS in both cultivars (Figure 3a). Approximately 27.5 and 23.6% of the DEGs were commonly upregulated and downregulated in Annabelle and Camel, respectively. Each cultivar also exhibited a set of uniquely regulated DEGs, with about 10–15% of the upregulated and downregulated genes being specific to either Annabelle or Camel (Figure S7A). These findings suggest that while both cultivars share a core transcriptional response to HS, they also exhibit distinct regulatory patterns.

Gene Ontology (GO) enrichment analysis was performed on both the common and unique upregulated and downregulated DEGs (Figure S7B). As expected, many of the commonly upregulated genes were enriched in categories related to stress, such as *heat response*, *protein folding*, and *cellular response to hypoxia*. Conversely, the downregulated genes were enriched for pathways associated with *responses to biotic stresses* and *photosynthetic capacity* (Figure S7B).

DNA methylation within gene regions has been shown to influence gene expression. For example, methylation of promoter regions is typically associated with transcriptional repression, although in some cases it may enhance gene expression (Lei et al., 2015; Zhang et al., 2018). To explore the relationship between heat‐induced changes in DNA methylation and gene expression, we focused on differentially methylated region‐associated DEGs (DMR DEGs). These were defined as DEGs overlapping with heat‐induced DMRs located within 2 kb upstream or downstream of the gene body. Among the 7904 (Annabelle) and 7542 (Camel) heat‐regulated DEGs, only 30 and 5, respectively, were identified as DMR DEGs (Tables S2, S6, and S7). Interestingly, the five DMR DEGs identified in Camel were also found in Annabelle.

Among these, two notable examples were identified, a *DNAJ* gene (*Soltu.DM.02G017060*) and an *HSP70* gene (*Soltu.DM.10G020640*) (Tables S6 and S7). The *DNAJ* gene was hypermethylated in the CHH context within its gene body and upregulated following HS, while the *HSP70* gene was hypomethylated in the CHG context upstream of the gene and downregulated.

The degree of DNA methylation is likely to be controlled by the relative expression/activity of DNA methyltransferases and DNA demethylases. Dutta et al. (2022) described the cytosine‐5 DNA methyltransferase (*C5‐MTase*) and demethylase (*DeMet*) gene families in potato and their tissue‐specific expression under control and HS conditions (Dutta et al., 2022). In agreement with their report, we found differential expression patterns of *C5‐MTases* and *DeMets* in potato leaves following HS (Figure S8). Interestingly, three *DeMets* encoding genes were significantly downregulated in both Annabelle and Camel during HS (Figure S7C). Homology search revealed that these DNA demethylase (*StDeMet1‐3*) genes are homologs of the *Arabidopsis thaliana Repressor of Silencing 1* (*AtROS1*) gene. AtROS1 has been shown to play an important role during HS in Arabidopsis. Its expression is inhibited by DNA demethylation of the *AtROS1* promoter, preventing heat‐induced activation of transposons by inhibiting their demethylation (Fan et al., 2025). Having seen downregulation of *AtROS1* homologous genes during HS in potato, we were curious to see whether this would correlate with demethylation of their promoter regions. However, there were no significant methylation changes between samples taken before or after HS for both cultivars (Figures S11– S16). This indicates that potato *ROS1* expression is likely not inhibited by demethylation as it has been shown for *A. thaliana*.

Given the very limited number of DMR‐DEGs identified relative to the total number of heat‐responsive DEGs (Table S5), we conclude that induced DNA methylation is unlikely to play a major direct regulatory role in heat‐responsive changes in gene expression in potato.

### Identifying constitutive DMR‐associated DEGs

Given that the majority of HS related transcriptional changes are unlikely to be explained by changes in heat‐induced DNA methylation and therefore are unlikely to account for the observed heat‐induced phenotypic differences, we considered an alternative explanation. Specifically, we hypothesized that constitutive differences in DNA methylation and gene expression between the two potato cultivars might underlie their differential phenotypic responses to HS. To investigate this, we compared the baseline transcriptomes of Annabelle and Camel, along with the previously identified constitutive DMRs at 28 days after planting, prior to the onset of HS.

Differential gene expression analysis prior to HS was conducted to identify genotype‐specific transcriptional differences. In total, 2972 DEGs were identified, comprising 1332 upregulated and 1640 downregulated genes (Figure 3b). These results suggest substantial constitutive variation in both gene expression and DNA methylation between the two cultivars (Table 1).

To assess whether constitutive transcriptional variation is associated with differences in DNA methylation, we identified DEGs that overlapped with constitutive DMRs between Annabelle and Camel, namely the DMR DEGs. These DMRs were located within 2 kb upstream or downstream of the gene body, the gene body and covered all methylation contexts (CG, CHG, and CHH). Of the 2972 DEGs, approximately 1786 genes (60.1%) were classified as DMR DEGs (Tables S3, S4, and S8). Most of these DMR DEGs were gene body DMR DEGs and found predominantly in the CG methylation context. This suggests that constitutive transcriptomic variation, when linked to methylation, is more likely to occur in CG‐methylated gene body regions.

GO enrichment analysis was performed to identify biological pathways enriched among DMR DEGs across different methylation contexts and genomic regions (Figure 3c; Table S9). We observed that DMR DEGs located within gene bodies—particularly those in the CG and CHG contexts—were significantly enriched in pathways related to biotic stress responses. For gene body DMR DEGs in the CG context, enriched GO terms included *response to jasmonic acid*, *defense response*, *plant‐type hypersensitive response*, and *sesquiterpene biosynthesis*. Similarly, gene body DMR DEGs in the CHG context were enriched in processes such as *response to bacterium*, *response to herbivore*, and *sesquiterpene biosynthesis* (Figure 3c; Table S9).

Interestingly, these findings mirror previous results comparing constitutive transcriptomic variation between heat‐tolerant and heat‐sensitive F1 progenies derived from Annabelle and Camel. In that study, the most prominent transcriptomic differences also involved genes related to biotic stress responses and sesquiterpene biosynthesis (Yeo et al., 2025).

**Figure 4:**
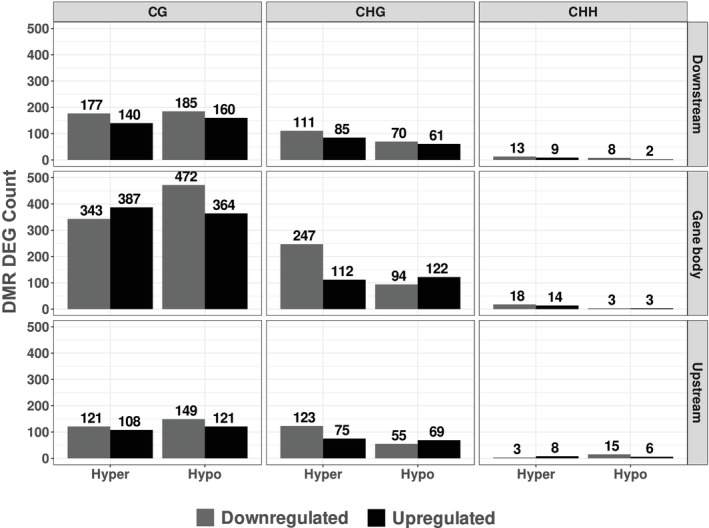
Summary of DMR differentially expressed gene (DEG) counts. Barplots of DMR DEG counts are separated in accordance with methylation context (CG, CHG, and CHH), genomic regions (Upstream, Gene body, and Downstream), methylation direction (Hypermethylated and Hypomethylated), and DEG regulation (Upregulated and Downregulated). The *Y* and *X*‐axes represent the DMR DEG counts and methylation direction, respectively. Hyper‐ or hypomethylation refers to the DMR DEGs being hypermethylated or hypomethylated in Annabelle relative to Camel. The color of the barplots represents if the DMR DEG is up‐ or downregulated. Up‐ and downregulation refers to DMR DEGs being upregulated or downregulated in Annabelle relative to Camel.

Building on earlier studies showing that DNA methylation can repress or enhance gene expression depending on the context (Lang et al., 2017; Lei et al., 2015; Zhang et al., 2018), we hypothesized that if DNA methylation functions as a widespread regulatory mechanism for gene expression across the potato genome, we expect to observe non‐random biased distributions of hyper or hypomethylated DMRs among up and downregulated DEGs. To test this, we categorized all constitutive DMR DEGs based on their methylation status (hyper‐ or hypomethylated) and expression pattern (up‐ or downregulated) (Figure 4; Table S3). We did not observe any strong bias in the distribution of methylation status relative to gene expression. Across gene body, upstream, and downstream regions, all DMR DEG types were relatively evenly distributed, with only minor deviations (within ~150 counts). There was, however, an exception for CHG within gene body and upstream DMR DEGs, where we observed a slightly unequal distribution. Within the DMR DEGs of these regions, there were more hypermethylated downregulated DMR DEGs and hypomethylated upregulated DMR DEGs as compared to the rest.

We then considered that some DEGs may contain more than one DMR across various regions (e.g., upstream and downstream simultaneously), potentially exerting a cumulative effect on gene expression (Figure S10). We analyzed how many of the 1786 unique DMR DEGs had multiple DMRs (termed ‘multi‐DMR DEGs’) and how these genes were expressed (Table S4). Of the 1786 unique DMR DEGs, 1040 had more than one DMR located in the gene body and/or flanking regions. Among these, 499 were unidirectional multi‐DMR DEGs (i.e., all DMRs were either hyper‐ or hypomethylated), while 541 were bidirectional (i.e., contained both hyper‐ and hypomethylated DMRs).

Focusing on the 499 unidirectional multi‐DMR DEGs, we examined whether methylation direction correlated with gene expression. Among these, 149 hypermethylated genes were downregulated, compared to 117 that were upregulated, a small difference that we consider biologically insignificant. Similarly, the number of hypomethylated unidirectional multi‐DMR DEGs was nearly identical between upregulated (117) and downregulated (116) genes (Table S4).

In conclusion, our findings suggest that overall constitutive differences in DNA methylation between potato cultivars are unlikely to play a major regulatory role in determining global gene expression.

### Constitutive DMRs are negatively associated with alternative allele frequencies

Since our analysis suggests that overall DNA methylation variation between potato cultivars is unlikely to regulate overall gene expression, we sought to explore other possible underlying reasons for this variation. Cytosines in the genome can undergo spontaneous deamination. Methylated cytosines deaminate more readily, resulting in T:G mismatches while unmethylated cytosine deamination results in U:G mismatches (Ehrlich et al., 1990; Wang et al., 1982). T:G mismatches are additionally less efficiently repaired compared to U:G mismatches. Consequently, methylated cytosines are more prone to mutations, specifically C to T transitions (Lutsenko & Bhagwat, 1999). Additionally, methylated cytosines have been shown to affect the mutability of neighboring nucleotides in vertebrates and plants (Chen et al., 2014; Kusmartsev et al., 2020; Qu et al., 2012).

**Figure 5:**
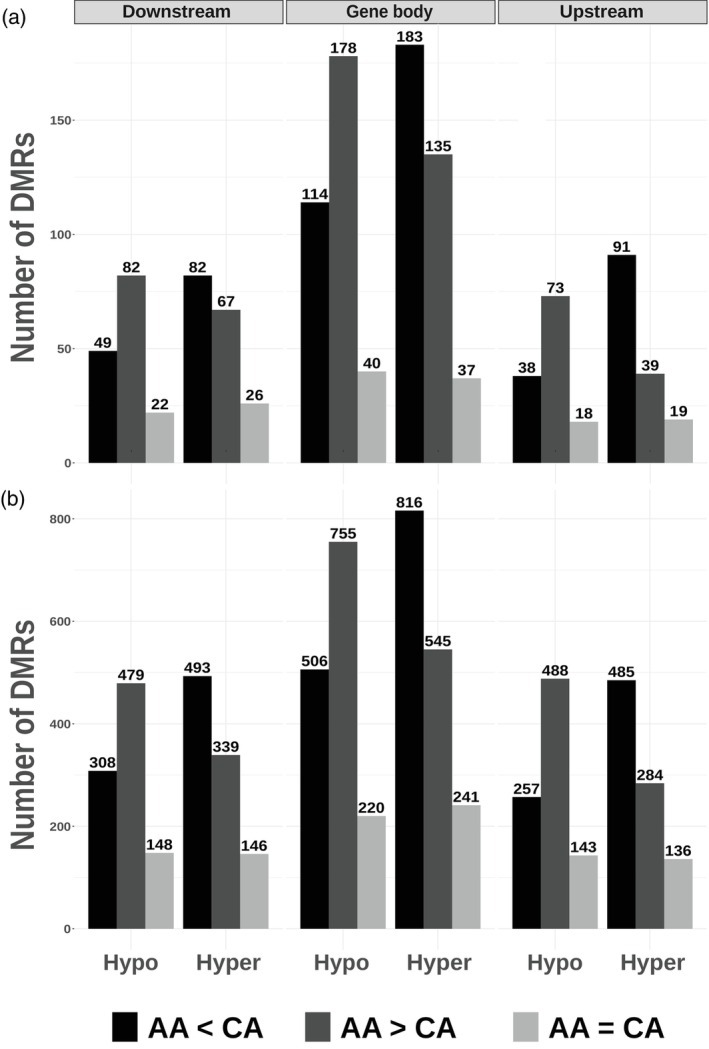
Summary of DMR count association with allele dosage differences between Annabelle (AA) and Camel (CA). Barplots showing the count distribution of DMRs on the *Y*‐axis that are separated by methylation direction (Hypermethylation and hypomethylation) on the *X*‐axis, allele dosage frequency (AA < CA, AA > CA, AA = CA) and genomic region (Upstream, Gene body and Downstream). The DMR counts represent the number of DMRs within each category, summarizing total hyper‐ and hypomethylated DMRs and their respective increased or decreased allele dosage frequency in AA relative to CA. (a) Summary of DMR counts with only DMR differentially expressed genes. (b) Summary of DMR counts with all DMR‐associated genes.

Whole genome sequencing (WGS) allelic variation was examined between Annabelle and Camel. We specifically assessed how allele dosage frequencies of identified SNPs correlate DMR‐associated genes and DEGs between the two cultivars. Among the DMRs associated with DEGs, hypomethylated regions in Annabelle relative to Camel were associated with increased allele dosages in Annabelle, whereas hypermethylated regions exhibited the opposite trend (Figure 5a). This pattern was consistent across upstream, gene body, and downstream DMR‐associated DEGs. A similar relationship was also observed across all DMR‐associated genes (Figure 5b).

To further explore this association, we focused on DMR‐associated genes containing C to T and T to C SNPs (and their reverse complements G to A and A to G), thereby accounting for cases in which the v6.1 *S. phureja* reference genome carries the alternative allele relative to the studied cultivars. This analysis allowed us to determine whether these SNPs, presumed to arise from the deamination of methylated cytosines, were also associated with DMR‐linked genes (Ehrlich et al., 1990; Wang et al., 1982). Consistent with previous observations, hypomethylated regions in Annabelle compared with Camel displayed higher allele dosages for these SNPs, whereas hypermethylated regions showed the opposite trend (Figure S9). Contrary to our initial expectations, these findings suggest that hypomethylation is generally associated with increased allele dosage, while hypermethylation corresponds to decreased allele dosage.

## DISCUSSION

### HS causes an overall hypermethylation in the potato methylome that does not affect overall heat‐mediated transcriptome changes

Abiotic stresses have been shown to induce changes in DNA methylation (Sun et al., 2022). In our study, we subjected the heat‐tolerant potato cultivar Annabelle and the heat‐sensitive cultivar Camel to HS at 37°C. We then sequenced the DNA methylome and transcriptome of the plants before (28 dap) and after (49 dap) the HS. Generally, DNA methylation density in potatoes is concentrated in repetitive regions, which aligns with findings in other plant species (An et al., 2017; Seymour et al., 2014; Shi et al., 2021). Our results revealed an overall hypermethylation of the potato DNA methylome after HS for both cultivars across all methylation contexts, except for CHG methylation in Camel. This minor hypermethylation appears to be primarily focused in repetitive genomic elements such as TEs rather than within gene features, which correlates with the downregulation of the three DNA demethylases discussed above (Figure S7C) after HS. Other studies have demonstrated that abiotic stresses in different plants, including mulberry, poplar, and rapeseed, can impact overall methylation levels through hyper‐ and hypomethylation processes (Li et al., 2020; Liang et al., 2014; Sun et al., 2022). For instance, DNA methylation in rapeseed under HS varies between heat‐tolerant and heat‐sensitive genotypes; heat‐tolerant genotypes tend to experience more hypomethylation events, while heat‐sensitive genotypes are more prone to hypermethylation (Gao et al., 2014).

In addition to global methylation changes induced by HS, we observed increased accumulation of TE transcripts in both potato cultivars, consistent with previous reports (Cavrak et al., 2014; Sun et al., 2020). In *A. thaliana*, the DNA demethylase ROS1 promotes TE activation by demethylating 2 kb flanking regions, particularly in the CHH context. Under HS, however, *ROS1* expression is suppressed through demethylation of its own promoter, thereby limiting its activity on TE loci and dampening TE activation (Fan et al., 2025).

In our study, the potato homologs of *ROS1* also showed reduced expression after HS. Unlike in Arabidopsis, we did not detect significant demethylation within 2 kb upstream of the potato *ROS1* homologs, suggesting that the heat‐induced repression of *ROS1* in potato is not regulated by DNA methylation. The reduction of *ROS1* expression may help explain the overall hypermethylation of TE bodies and their flanking regions observed in both cultivars after HS.

Nevertheless, despite this hypermethylation and reduced *ROS1* expression, TE transcripts still accumulated. This indicates that heat‐induced hypermethylation, particularly in the CHH context, may act as a compensatory mechanism to restrict, but not fully prevent, TE activation under stress conditions.

Consistent with the above observations, locally HS‐induced CHH DMRs were the most abundant among other methylation sequence contexts, and most of them were hypermethylated after HS, particularly for the HS‐tolerant cultivar Annabelle. This suggests that local CHH methylation changes contribute to the broader TE hypermethylation observed after HS. Similar observations have been described in other plant species such as rice and Arabidopsis (Dubin et al., 2015; Fan et al., 2025; Secco et al., 2015).

Although both potato cultivars displayed a general increase in DNA hypermethylation after HS, the number of newly induced DMRs was relatively small compared with the baseline differences already present between the two cultivars. In contrast, HS triggered extensive transcriptomic reprogramming, with 3400–4400 downregulated and upregulated DEGs detected in each cultivar. Only a small proportion of these DEGs overlapped with DMRs within a 2 kb window around the gene body, and notably, just two genes—homologs of *A. thaliana* HSPs—were consistently identified as both DEGs and DMRs in Annabelle and Camel.

This imbalance between widespread transcriptional shifts and limited methylation changes suggests that DNA methylation plays only a minor role in shaping the HS‐induced transcriptomic changes in potato. Our findings align with work in Arabidopsis, where DNA methylation has been shown to remain relatively stable under abiotic stress and is unlikely to serve as a primary driver of transcriptomic reprogramming (Ganguly et al., 2017, 2018; Secco et al., 2015). Similar evidence comes from rice, where DMRs associated with phosphate starvation were identified, yet transcriptional responses occurred prior to methylation changes, pointing to a limited regulatory contribution of DNA methylation under stress (Secco et al., 2015).

Together, these results reinforce the view that, in both model and crop plants, DNA methylation changes are not the primary mechanism driving rapid transcriptional responses to stress. Instead, they may function as a secondary or stabilizing layer of regulation.

### Constitutive DNA methylation and transcriptome variation between Annabelle and Camel

Most of the DMRs identified in our study were attributed to constitutive variation between the potato cultivars Annabelle and Camel, with a noticeable concentration in gene‐rich areas for CG and CHH contexts. Most of the constitutive DMRs were primarily found in the CG or CHG methylation contexts. Previous studies on plant species with well‐defined population structures based on SNP data have demonstrated that DNA methylation patterns, particularly in the CG context and partially in the CHG context, can reflect such structures (Galanti et al., 2022; Sammarco et al., 2024). This may explain the greater abundance of constitutive variations in CG and CHG DNA methylation compared to CHH. Additionally, we observed significant constitutive transcriptome variation between Annabelle and Camel. Of the DEGs, 67, 18.0, and 0.5% were classified as DMR DEGs within a 2 kb window of gene bodies for CG, CHG, and CHH contexts, respectively. GO enrichment analysis of the identified DMR DEGs revealed an overrepresentation of GO terms related to defense responses, responses to jasmonic acid, and terpenoid biosynthesis pathways, particularly within the DMR DEGs located in gene bodies. This suggests that the majority of constitutive DMR DEGs between Annabelle and Camel are related to responses against biotic stresses. Furthermore, previous reports from our group indicated that transcriptome variation between selected heat‐tolerant and heat‐sensitive F1 hybrids of Annabelle and Camel enriched in defense‐related genes and terpenoid biosynthesis genes (Yeo et al., 2025).

DNA methylation has been proposed to play a crucial role in directly influencing gene expression (Lei et al., 2015; Zhang et al., 2018). If this holds true in potato, we would expect to see a biased distribution of DMR DEGs, in particular more hypermethylated downregulated and hypomethylated upregulated DMR DEGs, especially within upstream regions. However, this was not observed; most types of DMR DEGs, as shown in Figure 4 and Table S3, appeared to be relatively evenly distributed across the various methylation contexts with minor deviations. CHG gene body and upstream DMR DEGs were exceptions, where we observed higher counts of hypermethylated downregulated DMR DEGs and hypomethylated upregulated DMR DEGs. This distribution of counts in CHG upstream DMR DEGs corresponds well to what was proposed in the literature, where promoter methylation upstream of genes represses their expression (Zhang et al., 2018). However, we do not see the same distribution in CG or CHH upstream DMR DEGs. Additionally, we explored the possibility that unidirectional multi‐DMR DEGs might be necessary for regulating expression. Similarly, we found no significant differences in the numbers of up‐ and downregulated unidirectional multi‐DMR DEGs (Table S4). This led us to conclude that the overall constitutive variation in DNA methylation is unlikely to directly regulate gene expression; however, we cannot exclude the possibility that some genes are regulated by DNA methylation. For example, the potato tuberigen gene *SP6A* was identified as a DMR DEG in the CG methylation context, exhibiting hypomethylation 2 kb up‐ and downstream of its gene body in Annabelle compared to Camel (Figure S10). *SP6A* was also expressed at significantly higher levels in Annabelle compared to Camel, resulting in apparent yield differences (Figure 3a; Figure S4). Whether this observed differential expression of *SP6A* is a direct consequence of constitutive differential methylation, or if it is a result of other underlying factors, remains unclear.

### Constitutive DNA methylation variation is inversely associated with SNP allele dosage frequency between Annabelle and Camel

Our analysis suggests that constitutive DNA methylation variation is unlikely to directly regulate gene expression, though exceptions may occur. This raises the question of why DNA methylome differences exist between potato cultivars and why CG‐context variations are concentrated in gene‐rich regions (Figure 1). As mentioned earlier, DNA methylation patterns in the CG and partially in the CHG context exhibit well‐defined population structures that reflect the origins of plant ecotypes and are somewhat correlated with SNP patterns (Galanti et al., 2022; Sammarco et al., 2024). Methylated cytosines are known to be more susceptible to deamination (which results in T:G mismatches) compared to unmethylated cytosines (which result in U:G mismatches). T:G mismatch repair, compared to U:G mismatch repair, is much less accurate and likely to contribute to mutations (Qu et al., 2012; Supek et al., 2014; Zhou et al., 2020). Qu et al. (2012) further demonstrated in humans a 50% increase in SNP incidence when a neighboring CG methylation site (within 10 bp) is methylated. In contrast, a more recent study in humans using rare polymorphisms indicated that nucleotide bases within 3 bp of methylated CG sites exhibit a decreased frequency of mutations (Kusmartsev et al., 2020). Furthermore, the same study found that for rice and *A. thaliana*, methylated CG sites are associated with increased mutation frequencies (Kusmartsev et al., 2020). In our study, limited to two contrasting cultivars and based on correlation analysis, hypomethylated regions tend to associate with higher allele dosage frequencies, whereas hypermethylated regions showed the opposite trend. *SP6A* gene for instance demonstrate this trend. Annabelle was hypomethylated upstream and downstream of *SP6A* relative to Camel. Annabelle additionally showed higher alternative allele dosage frequencies compared to Camel within the identified DMRs (Figures S17 and S18), thus indicating sequence variation within the DMRs. Importantly, the majority of the identified genetic variations did not coincide with DNA methylation sites within the identified DMRs of *SP6A*. This pattern is consistent with Kusmartsev et al. (2020), who observed similar associations for rare SNPs in humans. However, they reported the opposite trend in rice and *A. thaliana*, where methylated cytosines correlated with increased SNP incidence.

The differing trends observed between humans and different plant species in the aforementioned study may stem from variations in the base excision repair (BER) pathway utilized in response to the T:G and U:G mismatches upon deamination, namely the short‐patch (SP‐BER) and long‐patch base excision repair (LP‐BER) (Kusmartsev et al., 2020). For SP‐BER, a single nucleotide is inserted during repair while for LP‐BER, multiple nucleotides may be inserted during repairs (Córdoba‐Cañero et al., 2009). Although both types of BER have been reported in plants, it remains unclear which type repairs nucleotide mismatches, such as T:G or U:G, resulting in SP‐ or LP‐BER (Córdoba‐Cañero et al., 2009; Kusmartsev et al., 2020; Lee et al., 2014). Nevertheless, DNA BER has been documented to be linked with increased mutational burden on flanking regions of T:G and U:G mismatches that result from deamination. Chen et al. (2014) intentionally introduced mismatches that are normally repaired by BER into a SV40 episome capable of replication in human cells to observe the mutational risks of BER. These mismatches include naturally occurring T:G and U:G mismatches as a result of cytosine deamination. While both T:G and U:G mismatches were faithfully repaired, the process also significantly increased the mutation frequency of neighboring nucleotides. The mutation frequency was, however, much higher for U:G mismatches compared to T:G mismatches (Chen et al., 2014). Although speculative, this might explain partially why our observed hypomethylated regions are associated with higher allele dosage frequencies. However, the precise mechanism behind the BER and how it is linked to mutational frequencies is still unclear in plants.

In a broader perspective, Meng et al. (2016) performed a population‐based study on natural Swedish *A. thaliana* accessions and investigated the association between DNA methylation variation, genetic variation, and gene expression. The study found many more expression traits associated with SNP variation than with methylation variation. Most traits linked to methylation were also correlated with SNPs, which led Meng et al. (2016) to conclude that the effects of DNA methylation are marginal compared to genetic variation. Consistent with our study, although constitutive DNA methylation variation overlaps with DEGs between the two potato cultivars, its impact on expression is likely limited compared to genetic variation.

## CONCLUSION

Our study provides valuable insights into DNA methylation patterns and their associations with gene expression in two contrasting potato cultivars. While heat‐induced DNA methylation showed limited correlation with heat‐responsive transcriptome changes, constitutive DNA methylation variations appear to reflect underlying genetic differences between the cultivars. Although most methylation differences may not directly influence gene expression, they could be indicative of genetic variations that indirectly impact gene function. By highlighting these patterns, our findings lay the groundwork for future studies to experimentally explore the interplay between DNA methylation, genetic variation, and gene regulation in potato.

## MATERIALS AND METHODS

### Plant material, cultivation and phenotyping

Plants (*S. tuberosum L*.) of the two potato cultivars Annabelle (AA, HZPC, 2001) and Camel (CA, STET Holland, 2013) were obtained from SaKa Pflanzenzucht GmbH (Windeby, Germany) and propagated in tissue culture (Erlangen, Germany). Plants were first planted in 0.1 L pots for 1 week, grown in P‐Type substrate (Hawita, Vechta, Germany), and were then repotted to 2.5 L pots filled with T‐Type substrate (Hawita, Vechta, Germany mixed with vermiculite). The plants were grown in climate chambers (Snijders Labs, Tilburg, Netherlands) for 28 days under control conditions (ambient temperatures 21°C/19°C and long‐day photoperiod 16 h/8 h; day/night). Subsequently, all plants were shifted to heat conditions (37°C/35°C) and grown there for 3, 7, 14, 21 or 28 days. At the indicated time points, three plants of each cultivar were shifted back to control conditions for 14 days. The 15 plants of each cultivar were independent clones and were labeled #1–#3 for each HS duration. All plants were harvested 87 days after planting (dap). Tuber weight (g) and tuber number (*n*) were measured (Figures S1 and S4).

### Leaf sampling for RNA and DNA isolation

Leaf samples were taken at midday (after 6 h of light), from two fully expanded source leaves (6th and 7th leaf from the top) at 28 days after planting (control) and at the indicated time points after the shift to HS conditions. The samples were snap‐frozen in liquid N_2_. At each time point, three independent replicates for each cultivar were collected, with each replicate corresponding to a single plant. In this publication, leaf samples, prior and 21 days after the application of HS were sequenced (Figure S2). The three identical clones of cultivar AA and CA that were sequenced are numbered from #1 to #3.

### RNA isolation

Total RNA was isolated according to Logemann et al. (1987) from 100 mg ground leaf material. The quality and quantity were measured with a ND‐1000 spectrophotometer (NanoDrop ND1000, PEQLAB Biotechnologie GmbH, Erlangen, Germany). RNA samples were sent to Novogene GmbH, Munich, Germany on dry ice for RNA sequencing analysis.

### DNA isolation

Approximately 100 mg frozen and ground leaf material of each sample was sent to Novogene GmbH, Munich on dry ice. DNA isolation and further processing was done at Novogene for WGBS and WGS.

### RNA sequencing analysis

RNA sequencing reads were processed according to Yeo et al. (2025). TE expression was detected with the TEtranscripts software using identified repetitive features from v6.1 *S. phureja* reference genome (Jin et al., 2015).

### WGBS and WGS sequencing analysis

WGBS and WGS raw sequencing reads were first checked for quality using the FastQC software (v0.11.9). Sequencing reads with Phred quality base calls <30, read length <25 bp and reads with adapter content were discarded and trimmed using BBDuk from BBMap software (v39.01).

The trimmed WGS reads were then aligned to v6.1 *S. phureja* reference genome (https://phytozome‐next.jgi.doe.gov/) (Pham et al., 2020) using BWA‐MEM (v0.7.17‐r1188) with default parameters. Variant calling was performed using Freebayes (v0.9.21) with v6.1 *S. phureja* reference genome as the reference. Key parameters specified were ‐‐ploidy 4 ‐‐min‐mapping‐quality 20 ‐‐min‐base‐quality 20; the rest were left to default. Subsequently, the variant call format (VCF) file was filtered to retain only biallelic SNPs with a minor allele frequency above 0.1, with a quality score (QUAL) ≥30, read depth between 10 and 50.

The WGBS reads additionally had their first 10 nucleotides forcibly trimmed using the ftl parameter to avoid the possibility of methylation bias as a result of the sequencing. The reads were checked for quality again using FastQC. The filtered and trimmed WGBS reads were aligned to the v6.1 *S. phureja* reference genome (https://phytozome‐next.jgi.doe.gov/) (Pham et al., 2020) using the Bismark (v0.24.1) software (Krueger & Andrews, 2011). Duplicated WGBS reads were removed using the Bismark deduplication function as recommended by the software. The methylation level and the coverage of each methylation site were determined and called using the bismark_methylation_extractor function with the genome‐wide cytosine report parameter to generate CX files for further analyses. Metagene methylation plots of genomic features according to v6.1 *S. phureja* reference genome and methylation levels calculated at chromosome levels were performed in python using the BSXplorer (v1.0.1) library (Yuditskiy et al., 2024). DMRs for each cytosine context were identified using the R DMRcaller (v1.36.0) package using the ‘noise_filter’ method and ‘gaussian’ kernel function (Catoni et al., 2018). DMRs were only considered significant if the FDR <0.05, minimum coverage per cytosine was 8, DMR size was 50, 50 and 25 bp for CG, CHG and CHH, respectively, minimum proportion of difference is 40, 30 and 10% for CG, CHG and CHH context, respectively. The rest of the parameters of the DMR caller were left to default. Visualization of DNA methylation levels was generated in the integrated genomics viewer (IGV) from bismark bedGraph files.

DMR‐associated genes, DEGs, and SNPs overlaps were identified using bedtools (v2.29.2) within upstream and downstream regions of gene body using a window of 2 kb. This step in some instances identified more than one DMR (minimum DMR gap size = 200 bp) that were associated with a given gene, which could be either hyper or hypomethylated and were considered in our analysis. Circos plots of the density of genomic features, chromosome‐level methylation density, and DMR density were created using the R package circlize (v0.4.16). For the correlation analysis between DMRs and allele variation between Annabelle and Camel, we first accounted for the autotetraploid nature of potato, namely that each biallelic SNP locus theoretically contains up to four copies of an allele (SNPs were called by Freebayes with a ploidy = 4). Accordingly, the term allele dosage represents the number of copies (from 0 to 4) of the alternative allele (or non‐reference allele). The total SNP allele dosage for each DMR was calculated by summing the allele dosages of all SNPs that are located within that particular DMR for both cultivars. The resulting dosage frequency reflects the extent to which non‐reference alleles accumulate within each DMR in the two cultivars. Consensus sequences of *SP6A* from both cultivars, including 2 kb flanking regions, were generated from the VCF file using Bcftools (v1.14) and realigned to the v6.1 *S. phureja* reference genome with Minimap2 (v2.30‐r1287) for visualization in IGV. Unless stated otherwise, all other plots and analyses were performed in R.

## AUTHOR CONTRIBUTIONS

DSGY analyzed most of the data. JE and SS performed the heat stress experiments. JB performed the WGS alignment and variant calling. FK and JL provided the samples of Annabelle and Camel. AK contributed to the acquisition of WGS data. US supervised and designed the study. DSGY, JE, and US wrote the final manuscript. All authors edited and approved the final draft.

## CONFLICT OF INTEREST

The authors declare no conflict of interest.

## Supporting information


**Figure S1.** Experimental setup of the HS experiment with the potato cultivars Annabelle (AA) and Camel (CA).

**Figure S2.** Phenotypes of plants grown in HS for 21 days and after 14 days of recovery of Annabelle (AA) and Camel (CA).

**Figure S3.** Phenotypes of Annabelle (AA) and Camel (CA) grown in HS for different durations.

**Figure S4.** Harvest data of plants grown in HS conditions.

**Figure S5.** Methylation distribution frequency plot for all samples.

**Figure S6.** Overall TE features transcript abundance plot for all samples represented by *z*‐scores on the *Y*‐axis.

**Figure S7.** Differential gene expression analysis of Annabelle (AA) and Camel (CA) under HS.

**Figure S8.** Expression pattern of *C5‐MTases* and *StDeMets*.

**Figure S9.** Summary of DMR count association with allele dosage (C:T, T:C, G:A and A:G SNPs) differences between Annabelle (AA) and Camel (CA).

**Figure S10.** DNA methylation levels of *SP6A* gene (*Soltu.DM.05G026370*) within 5 kb flanking regions between Annabelle and Camel.

**Figure S11.** DNA methylation levels of *ROS1* gene (*Soltu.DM.11G005260*) within 5 kb flanking regions of Annabelle.

**Figure S12.** DNA methylation levels of *ROS1* gene (*Soltu.DM.11G005260*) within 5 kb flanking regions of Camel.

**Figure S13.** DNA methylation levels of *ROS1* gene (*Soltu.DM.10G024770*) within 5 kb flanking regions of Annabelle.

**Figure S14.** DNA methylation levels of *ROS1* gene (*Soltu.DM.10G024770*) within 5 kb flanking regions of Camel.

**Figure S15.** DNA methylation levels of *ROS1* gene (*Soltu.DM.09G004240*) within 5 kb flanking regions of Annabelle.

**Figure S16.** DNA methylation levels of *ROS1* gene (*Soltu.DM.09G004240*) within 5 kb flanking regions of Camel.

**Figure S17.** Positions of DNA methylation sites (1) in Annabelle and Camel, the sequences (2) of Annabelle (blue bars) and Camel (red bars) and the v6.1 *S. phureja* reference sequence (3) within the DMR region (DMR 3) identified upstream of *SP6A*.

**Figure S18.** Positions of DNA methylation sites (1) in Annabelle and Camel, the sequences (2) of Annabelle (blue bars) and Camel (red bars) and the v6.1 *S. phureja* reference sequence (3) within the two separate windows (A and B) of the DMR region (DMR 1) identified downstream of *SP6A*.

**Table S1.** Total number of differentially expressed repetitive elements.
**Table S2.** Total number of induced DMR DEGs identified for Annabelle and Camel.
**Table S3.** Total number of constitutive DMR DEGs between Annabelle and Camel for each region.
**Table S4.** Total number of uniquely identified constitutive DMR DEGs.
**Table S5.** DEG enrichment result of before and after heat stress comparison.
**Table S6.** List of heat‐induced DMR DEGs for Annabelle.
**Table S7.** List of heat‐induced DMR DEGs for Camel.
**Table S8.** List of constitutive DMR DEGs between Annabelle and Camel.
**Table S9.** Enrichment results of identified DMR DEGs between Annabelle and Camel.

## Data Availability

The WGBS and RNAseq sequencing data of Annabelle and Camel were submitted to NCBI with the project number PRJNA1338108. The WGS sequencing data of Annabelle and Camel were submitted to NCBI with the project number PRJNA1337306.
