# Supplementary figures and images for: Genotype‐dependent DNA methylation patterns are negatively associated with allelic variation rather than heat‐induced gene expression in two contrasting potato genotypes
Source: Plant J. 2026 Jan 18;125(2):e70690. doi: 10.1111/tpj.70690 (PMC12812439; doi:10.1111/tpj.70690)

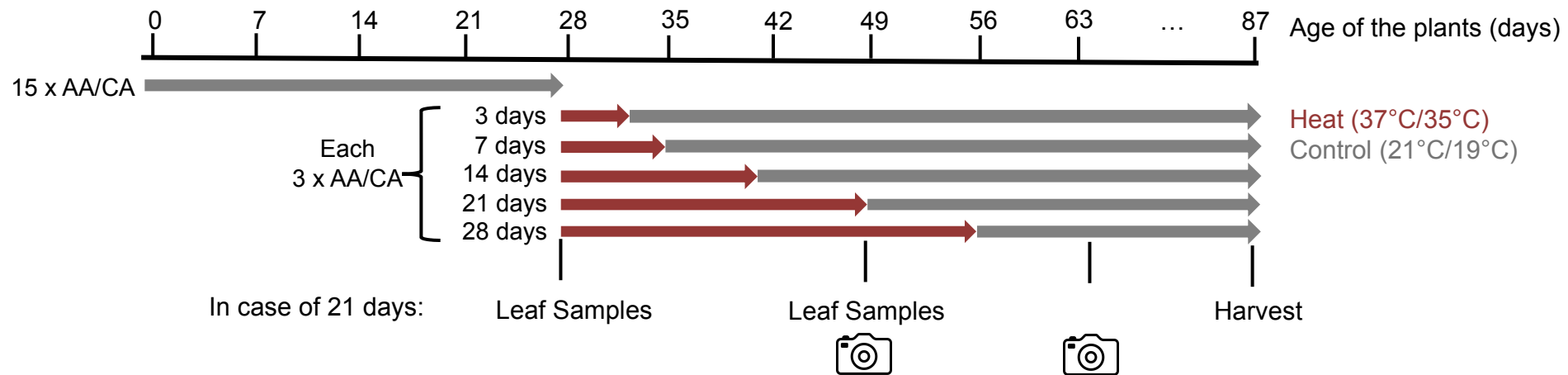

Supplement: Supplementary file 1 — Figure S1. Experimental setup of the HS experiment with the potato cultivars Annabelle (AA) and Camel (CA). [file TPJ-125-0-s016.pdf]

AA

CA

21 days:  
37°C

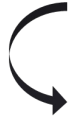

14 days:  
recovery

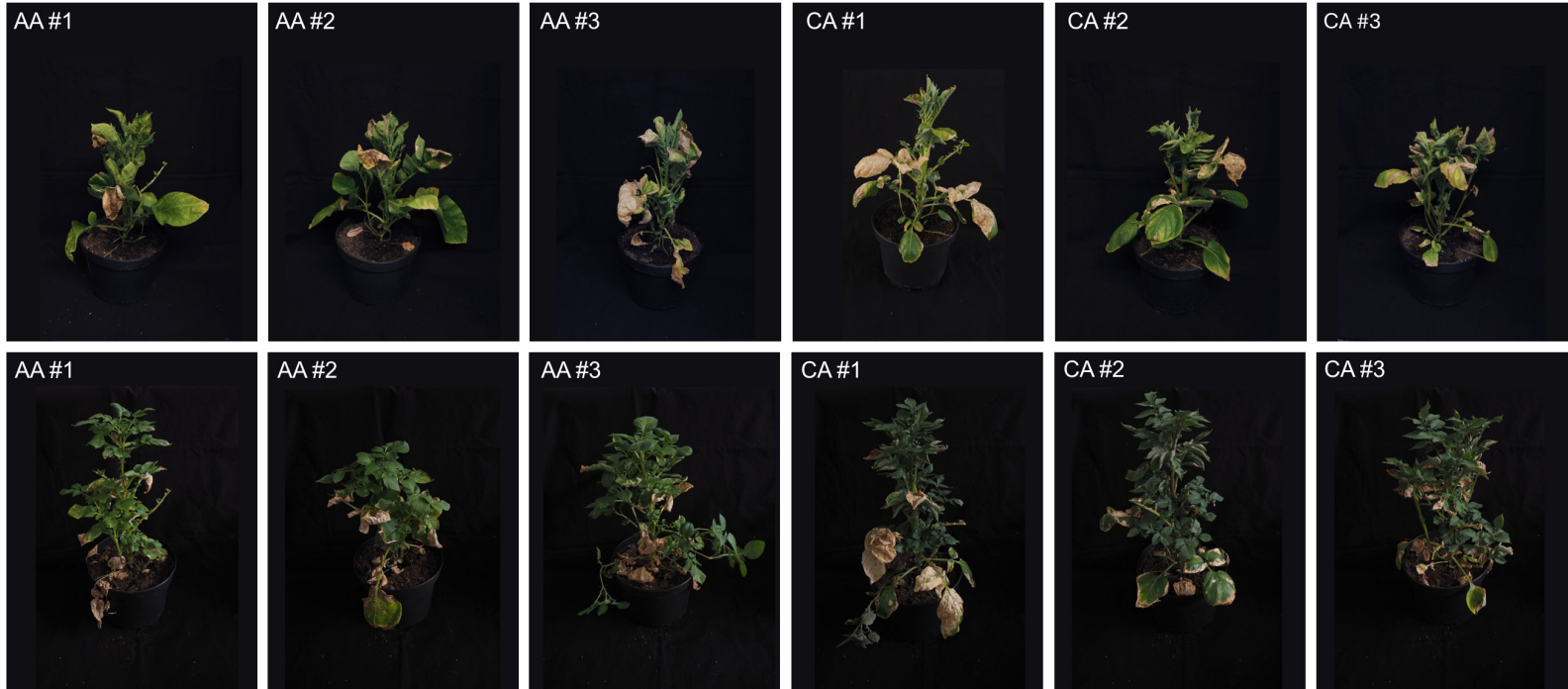

Supplement: Supplementary file 2 — Figure S2. Phenotypes of plants grown in HS for 21 days and after 14 days of recovery of Annabelle (AA) and Camel (CA). [file TPJ-125-0-s013.pdf]

A

14 days:  
37°C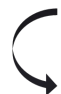14 days:  
recovery

AA

CA

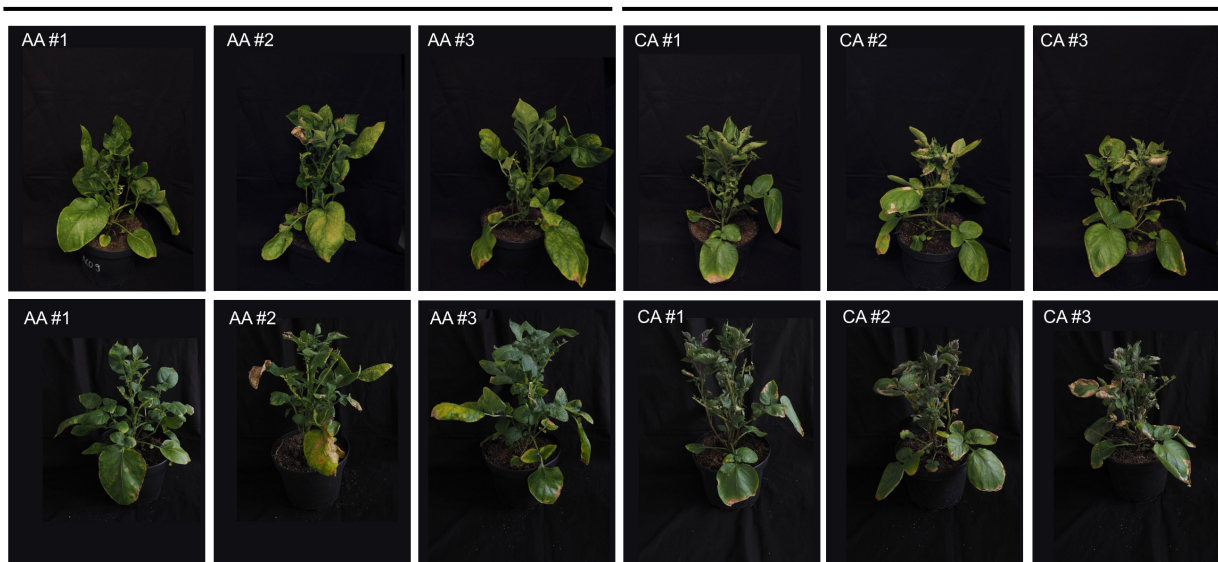

B

28 days:  
37°C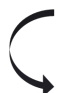14 days:  
recovery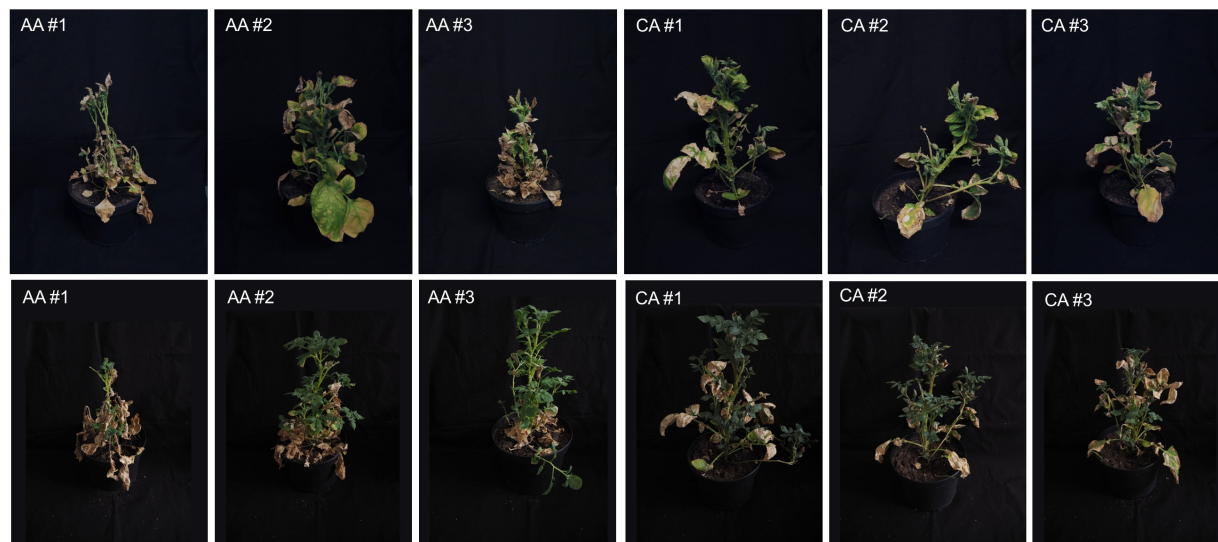

Supplement: Supplementary file 3 — Figure S3. Phenotypes of Annabelle (AA) and Camel (CA) grown in HS for different durations. [file TPJ-125-0-s008.pdf]

**A**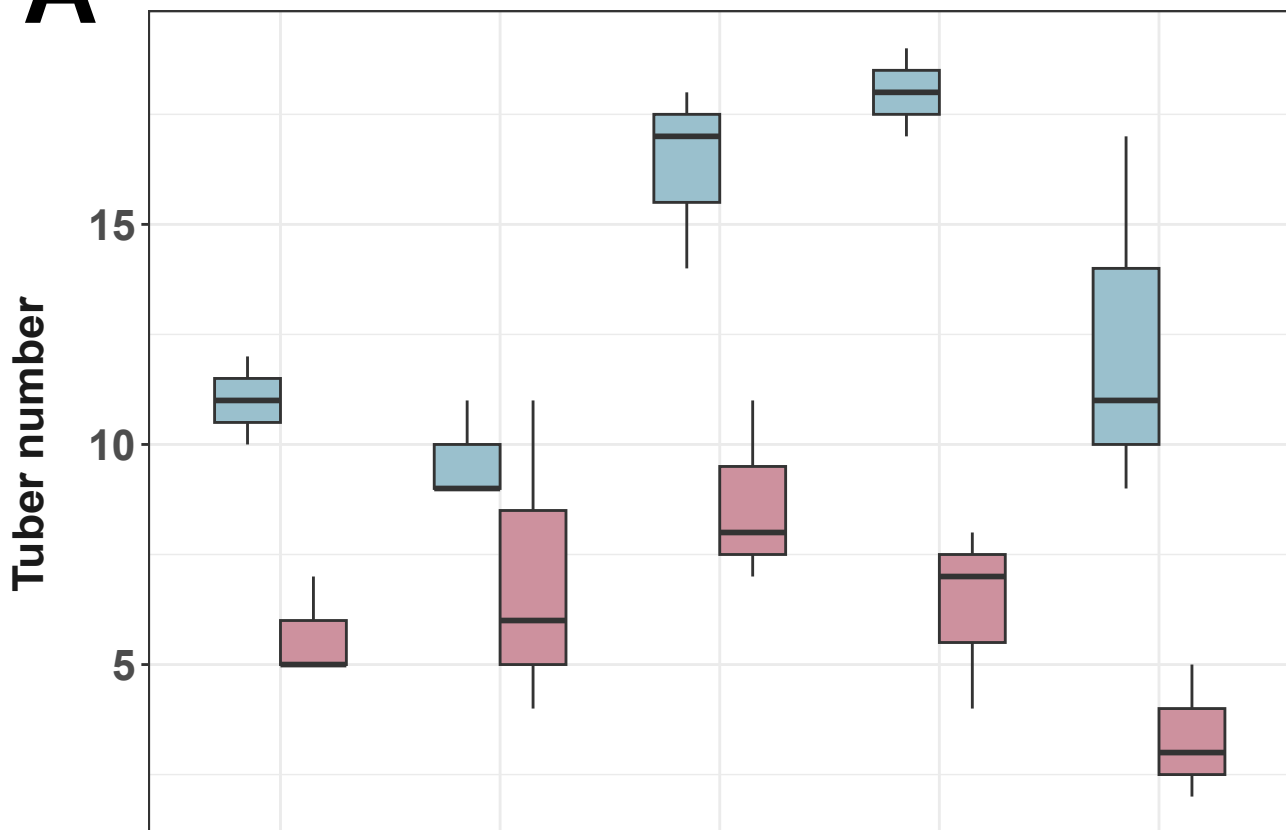**B**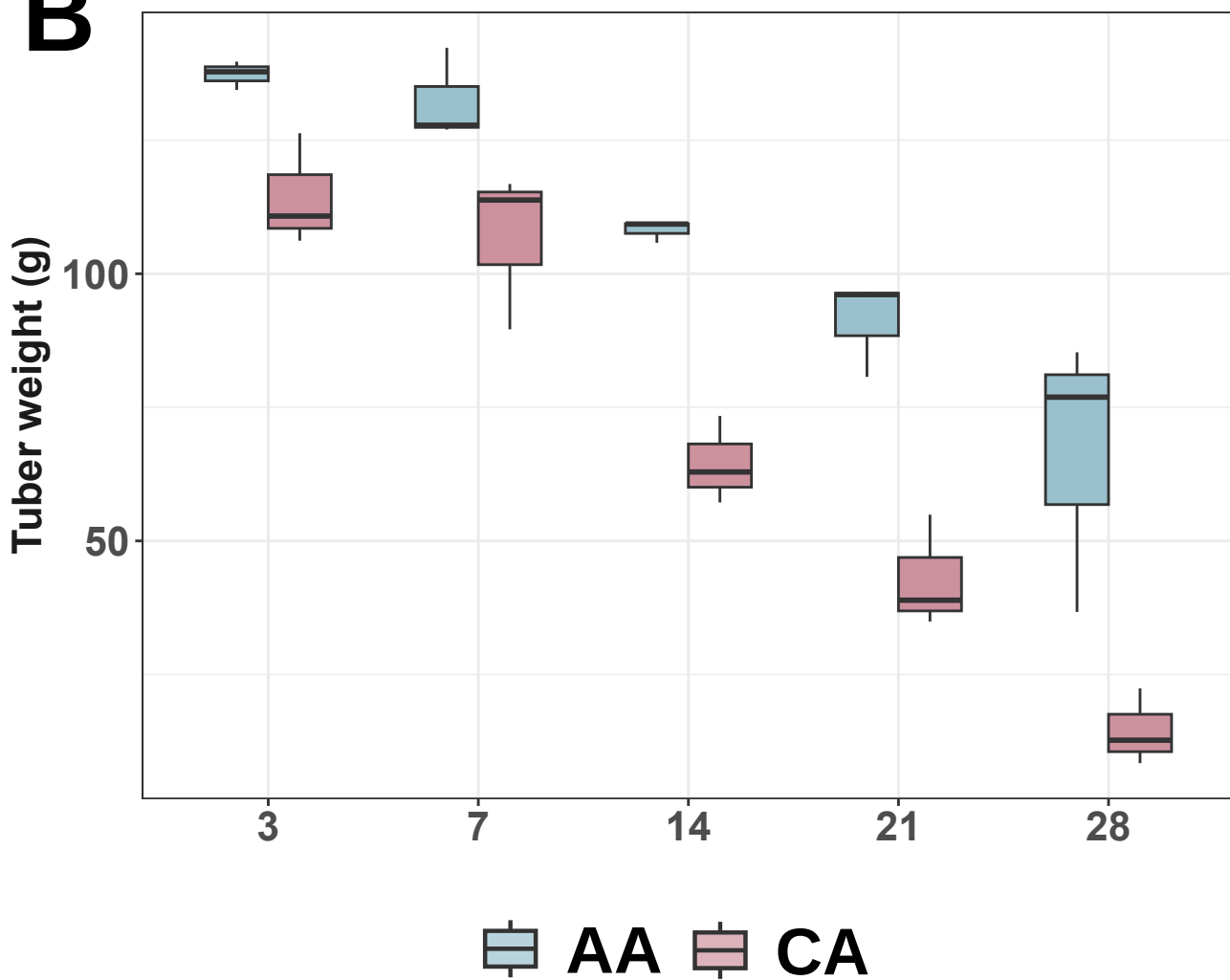

Supplement: Supplementary file 4 — Figure S4. Harvest data of plants grown in HS conditions. [file TPJ-125-0-s011.pdf]

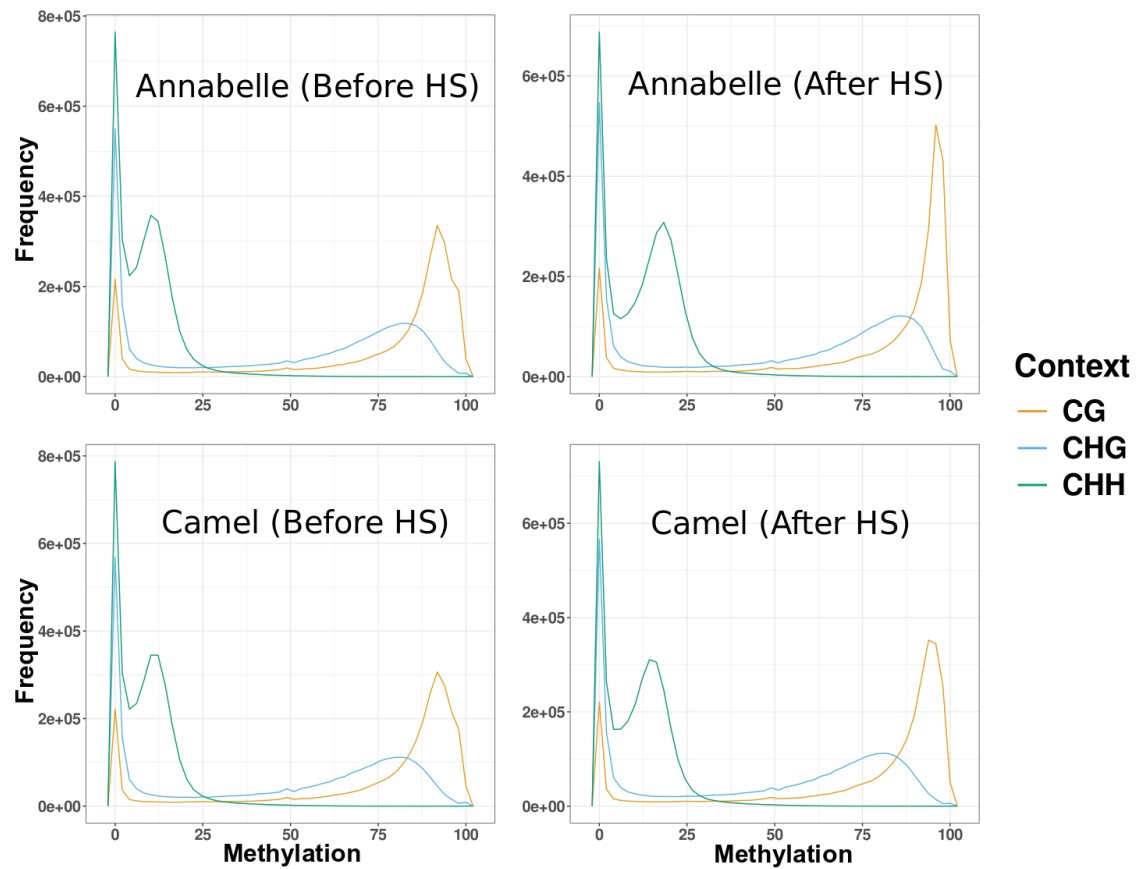

Supplement: Supplementary file 5 — Figure S5. Methylation distribution frequency plot for all samples. [file TPJ-125-0-s002.pdf]

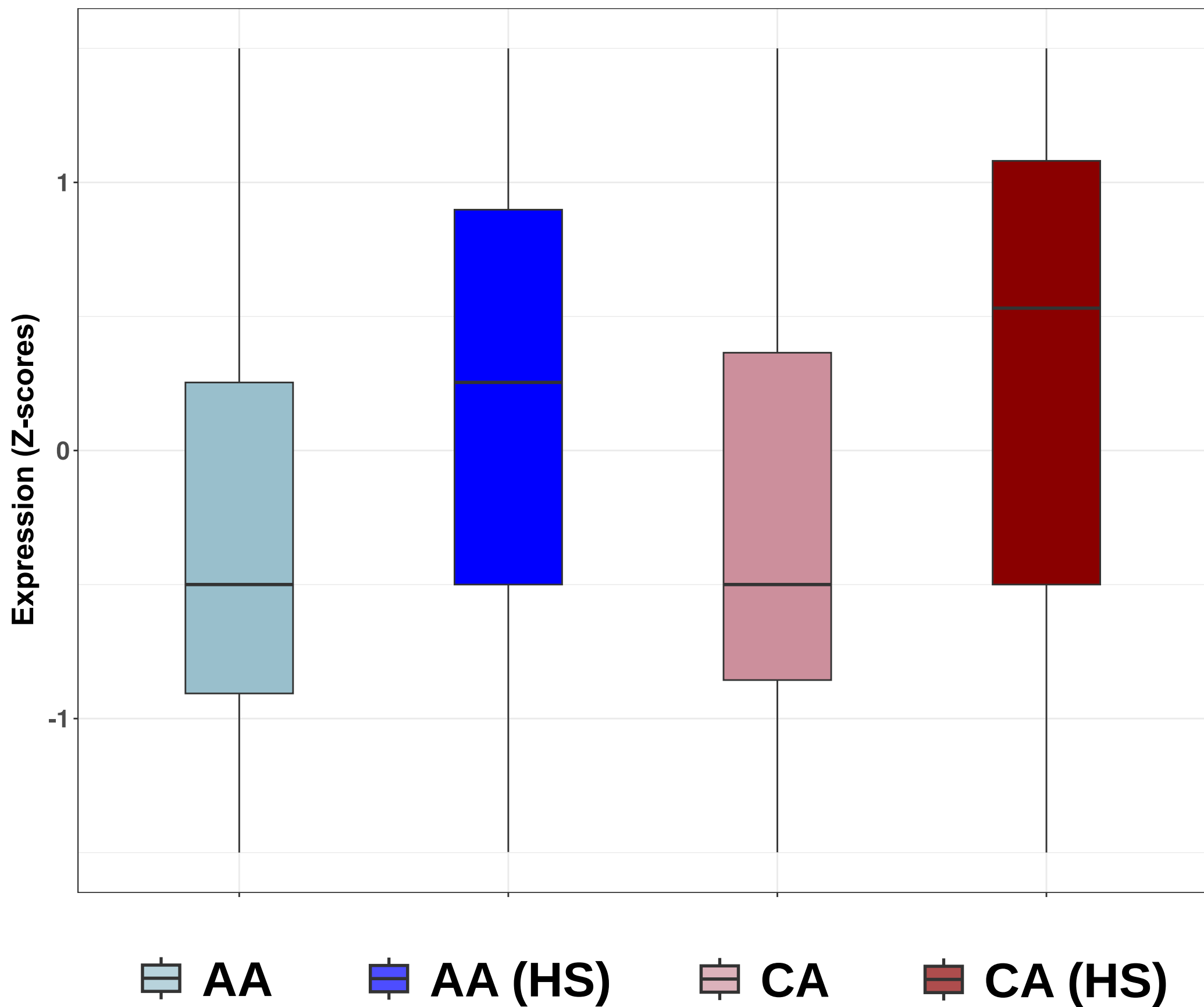

Supplement: Supplementary file 6 — Figure S6. Overall TE features transcript abundance plot for all samples represented by z‐scores on the Y‐axis. [file TPJ-125-0-s019.pdf]

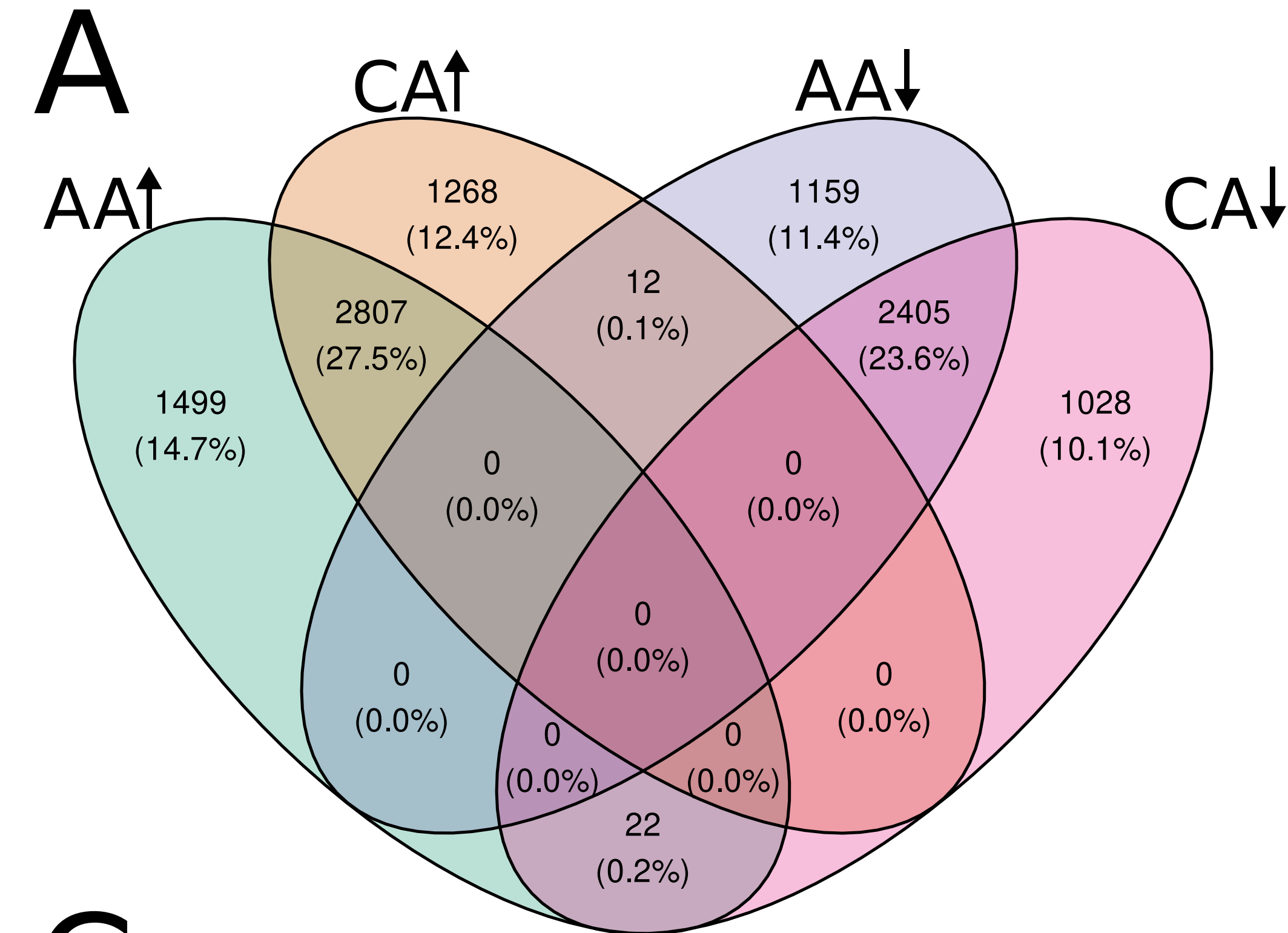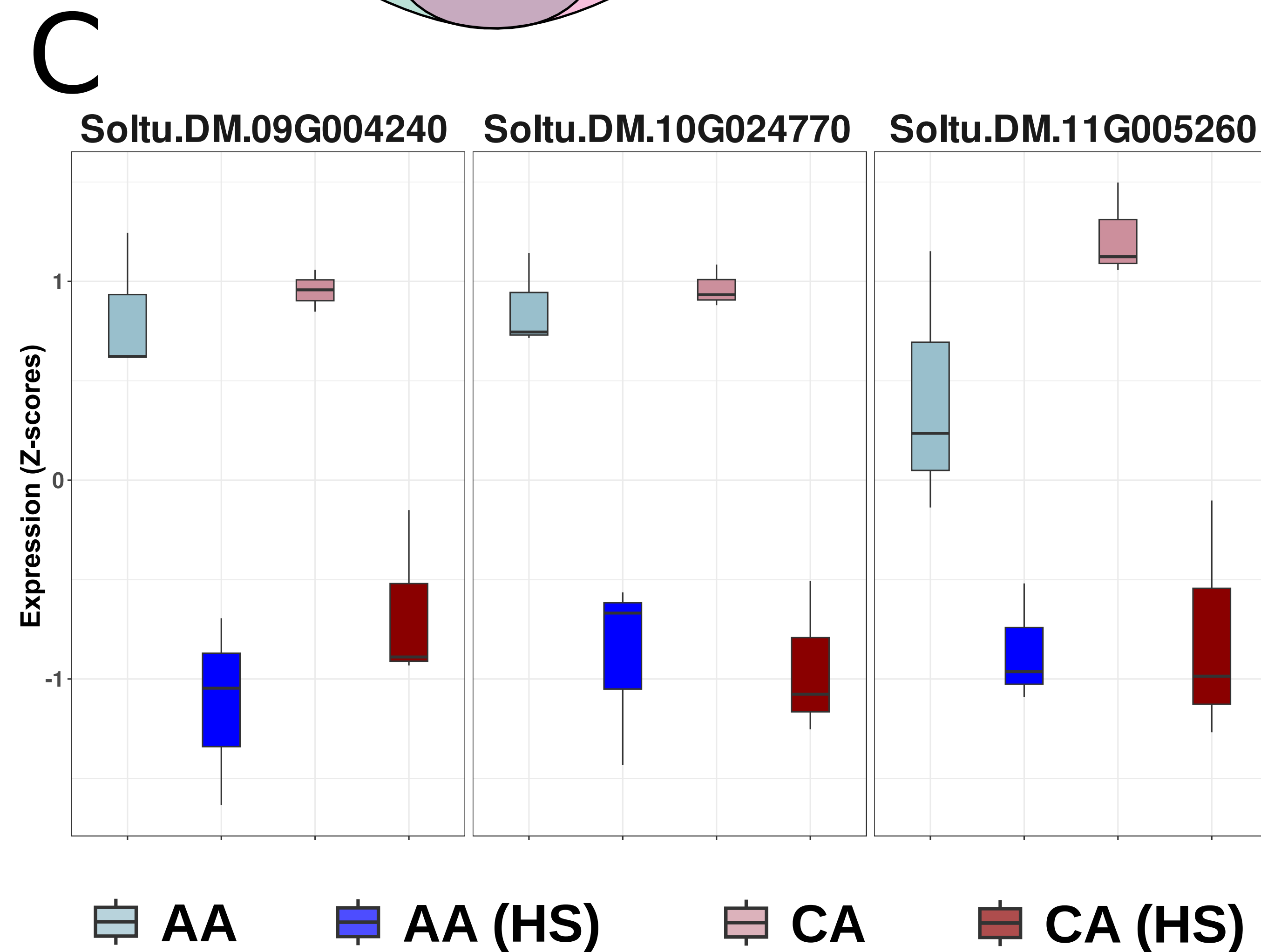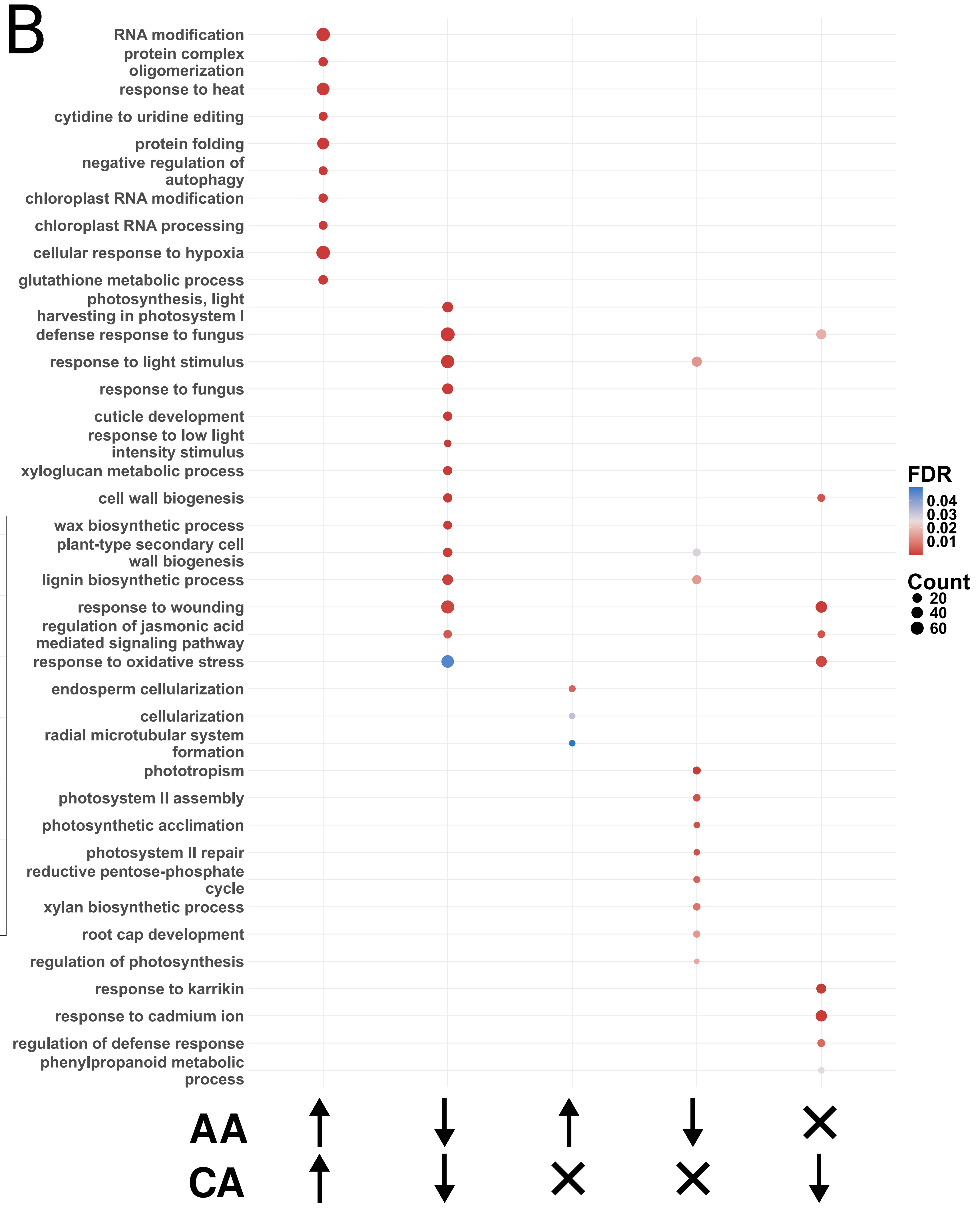

Supplement: Supplementary file 7 — Figure S7. Differential gene expression analysis of Annabelle (AA) and Camel (CA) under HS. [file TPJ-125-0-s018.pdf]

A

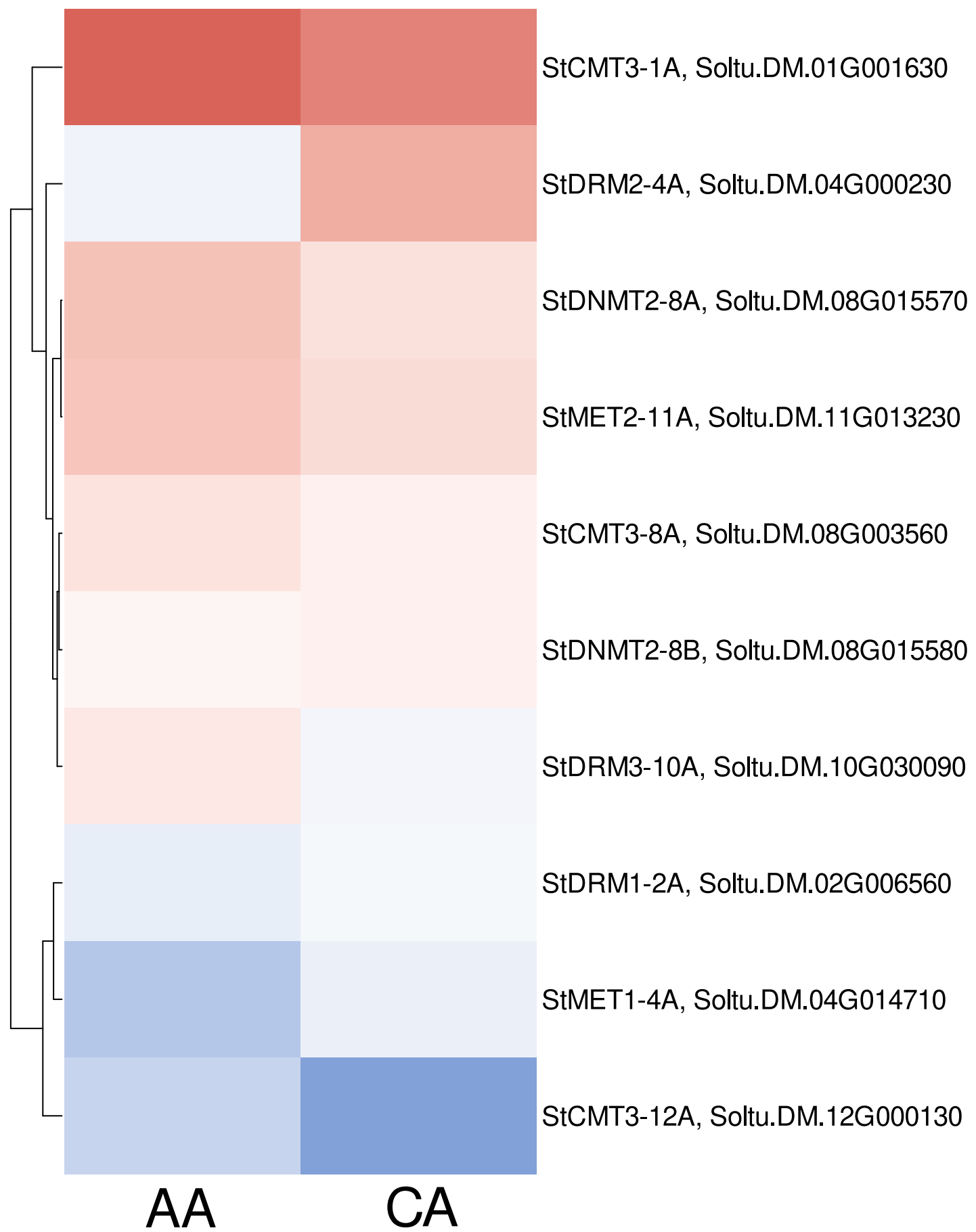

B

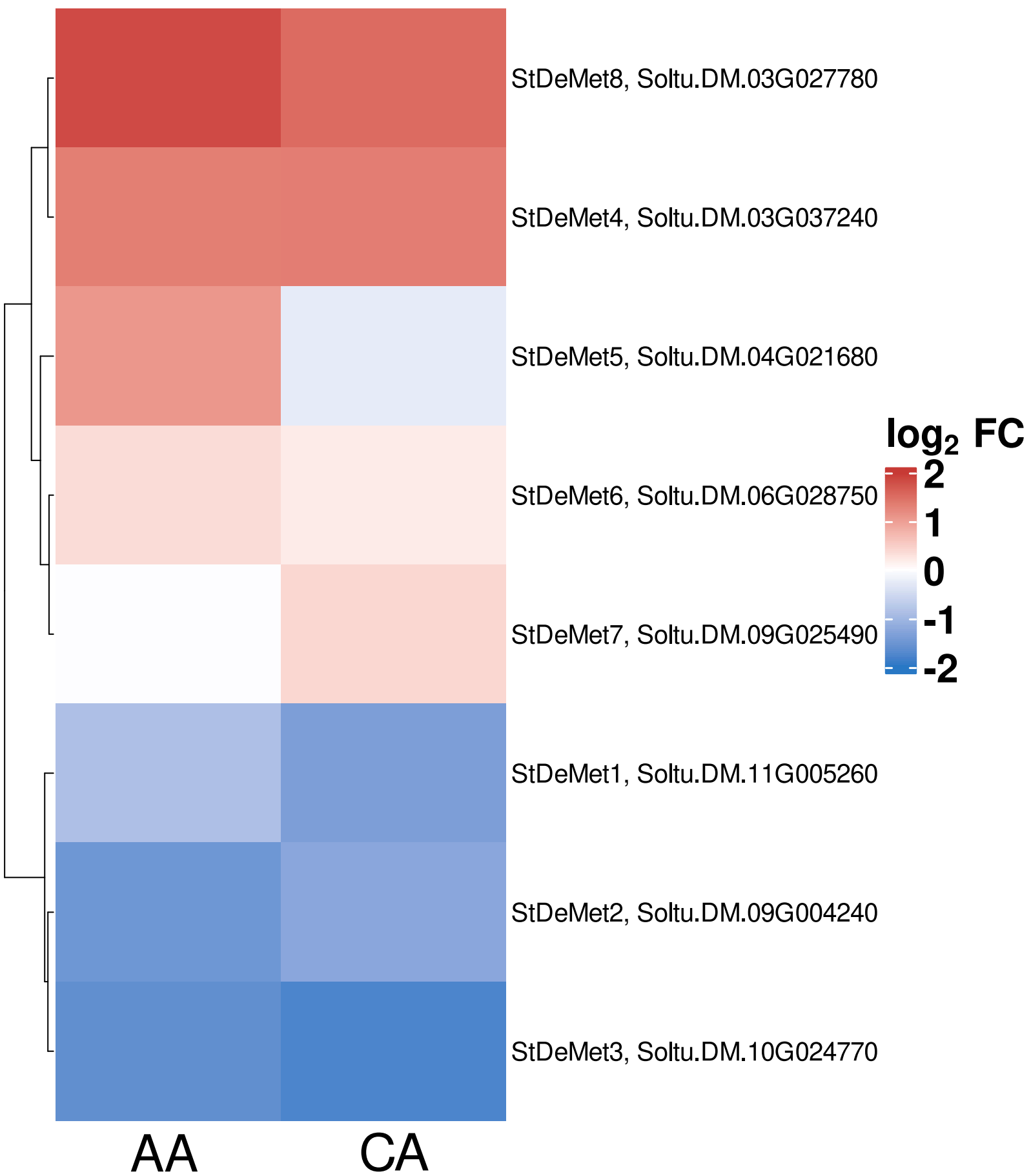

Supplement: Supplementary file 8 — Figure S8. Expression pattern of C5‐MTases and StDeMets. [file TPJ-125-0-s005.pdf]

Number of DMRs

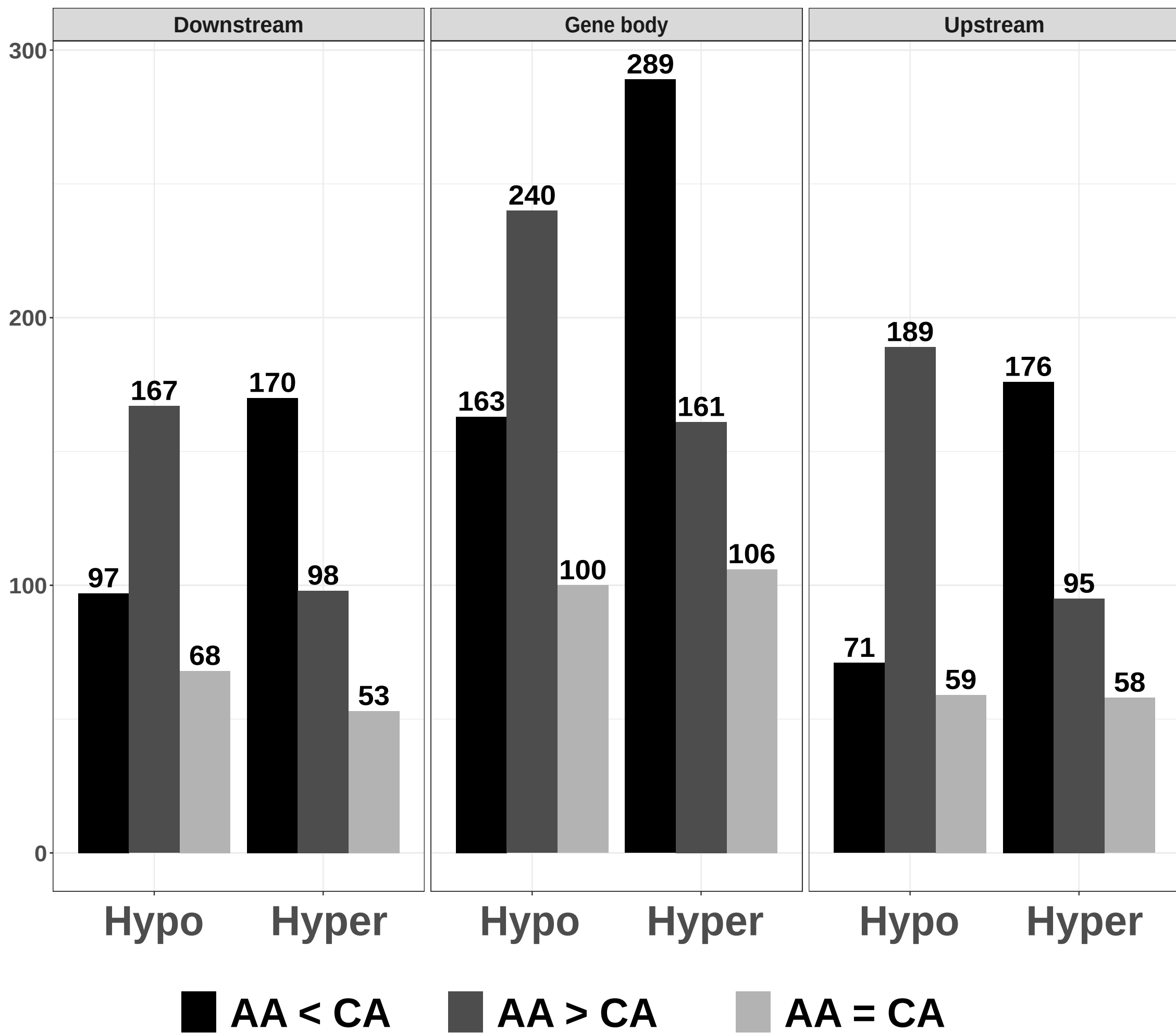

Supplement: Supplementary file 9 — Figure S9. Summary of DMR count association with allele dosage (C:T, T:C, G:A and A:G SNPs) differences between Annabelle (AA) and Camel (CA). [file TPJ-125-0-s009.pdf]

DMR 1

DMR 2

DMR 3

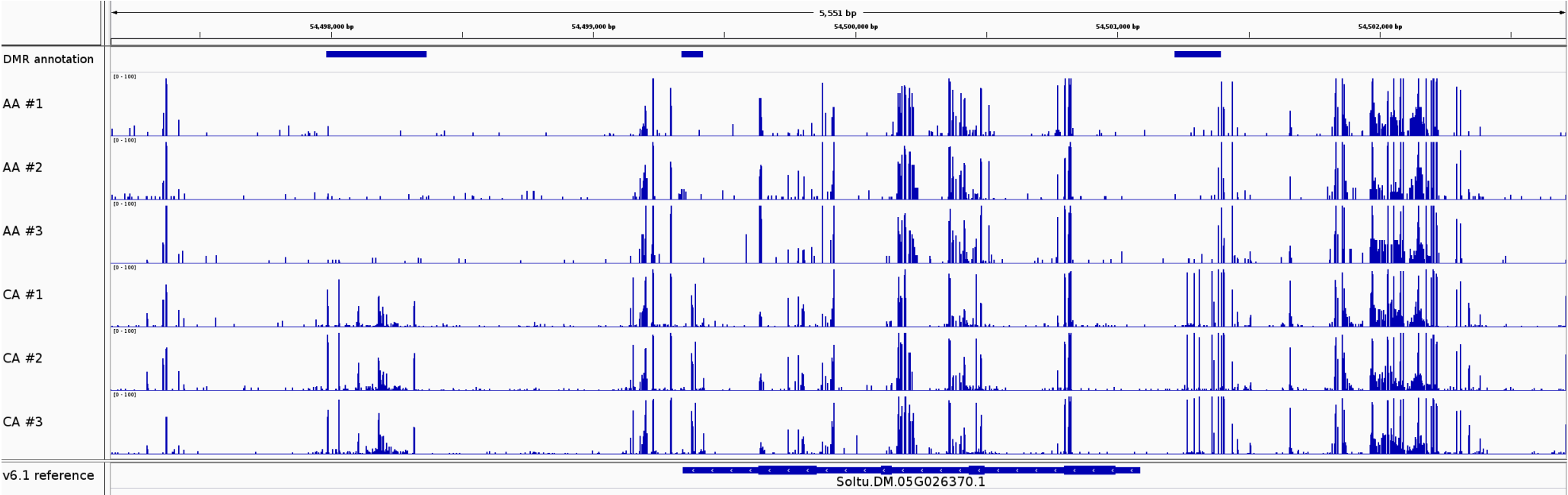

Supplement: Supplementary file 10 — Figure S10. DNA methylation levels of SP6A gene (Soltu.DM.05G026370) within 5 kb flanking regions between Annabelle and Camel. [file TPJ-125-0-s017.pdf]

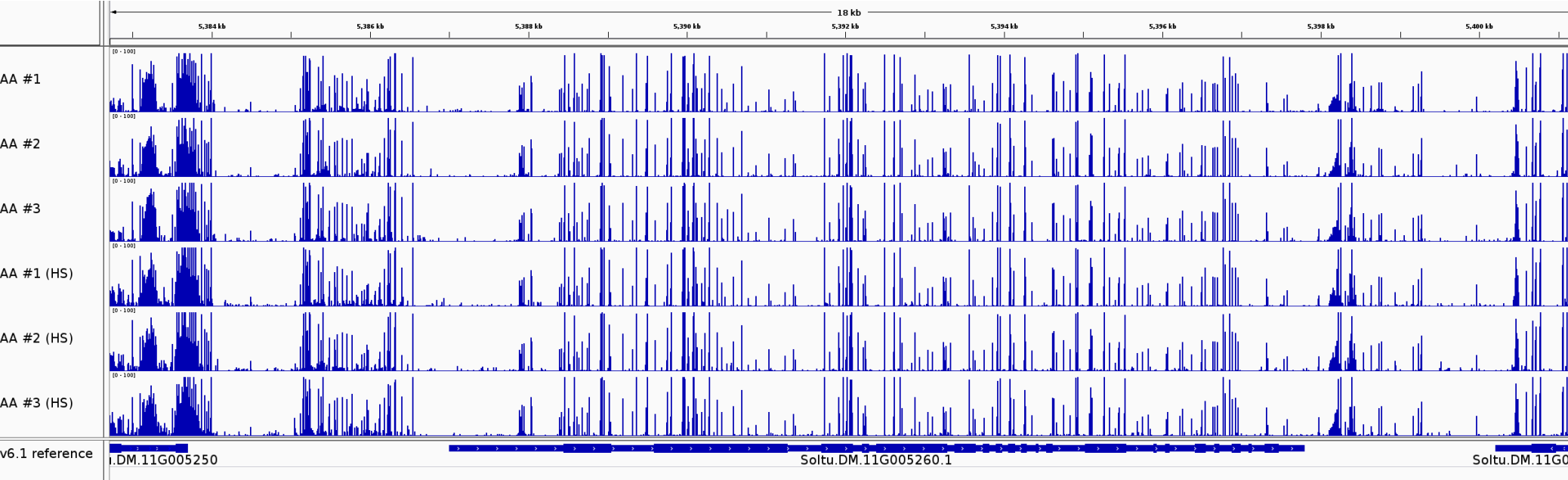

Supplement: Supplementary file 11 — Figure S11. DNA methylation levels of ROS1 gene (Soltu.DM.11G005260) within 5 kb flanking regions of Annabelle. [file TPJ-125-0-s001.pdf]

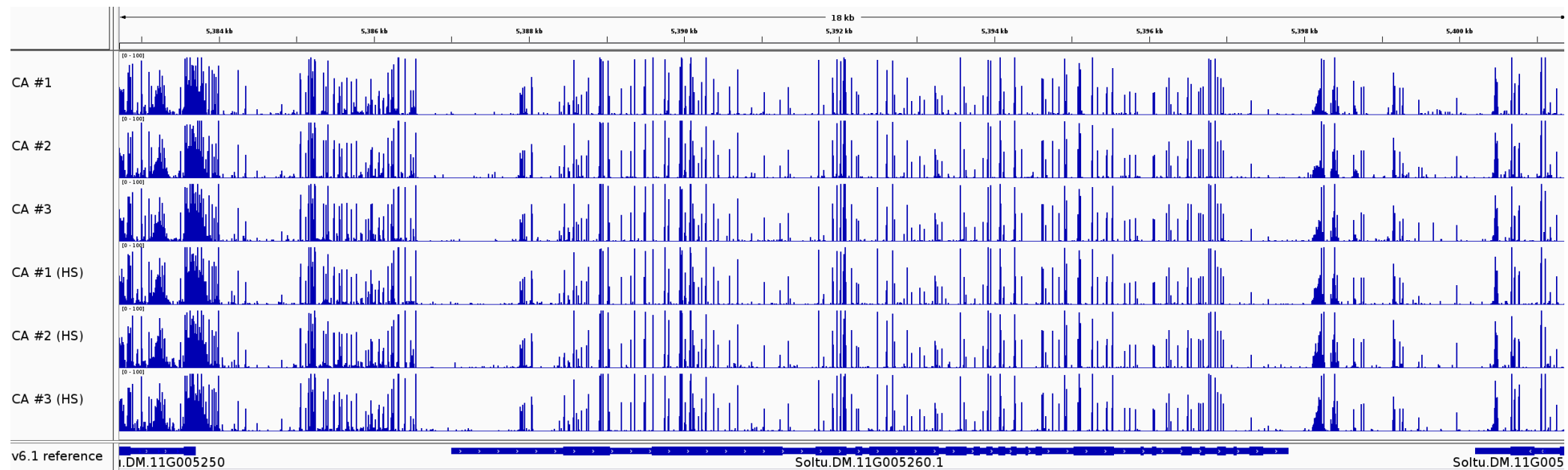

Supplement: Supplementary file 12 — Figure S12. DNA methylation levels of ROS1 gene (Soltu.DM.11G005260) within 5 kb flanking regions of Camel. [file TPJ-125-0-s004.pdf]

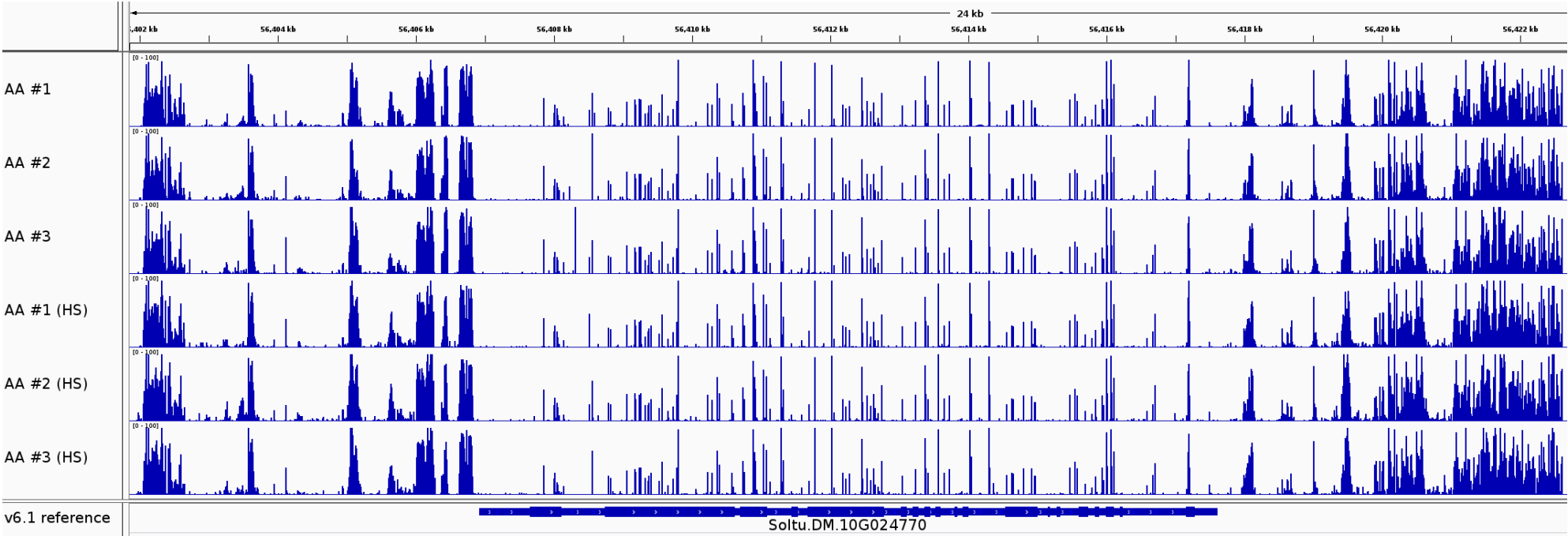

Supplement: Supplementary file 13 — Figure S13. DNA methylation levels of ROS1 gene (Soltu.DM.10G024770) within 5 kb flanking regions of Annabelle. [file TPJ-125-0-s014.pdf]

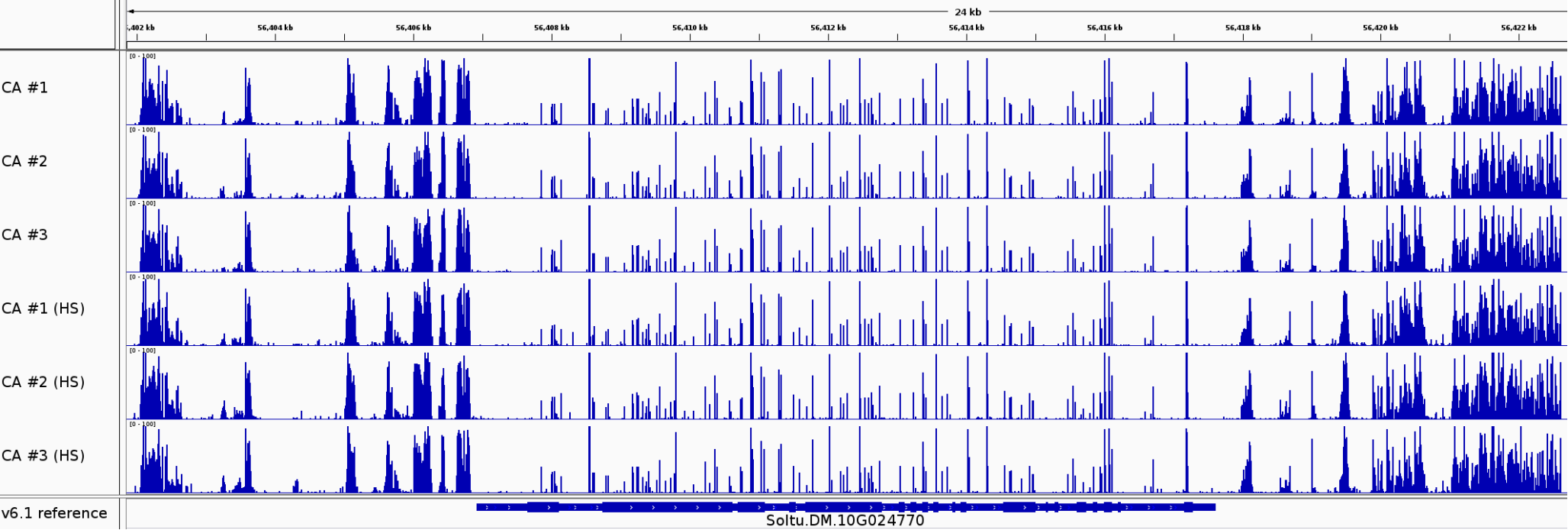

Supplement: Supplementary file 14 — Figure S14. DNA methylation levels of ROS1 gene (Soltu.DM.10G024770) within 5 kb flanking regions of Camel. [file TPJ-125-0-s015.pdf]

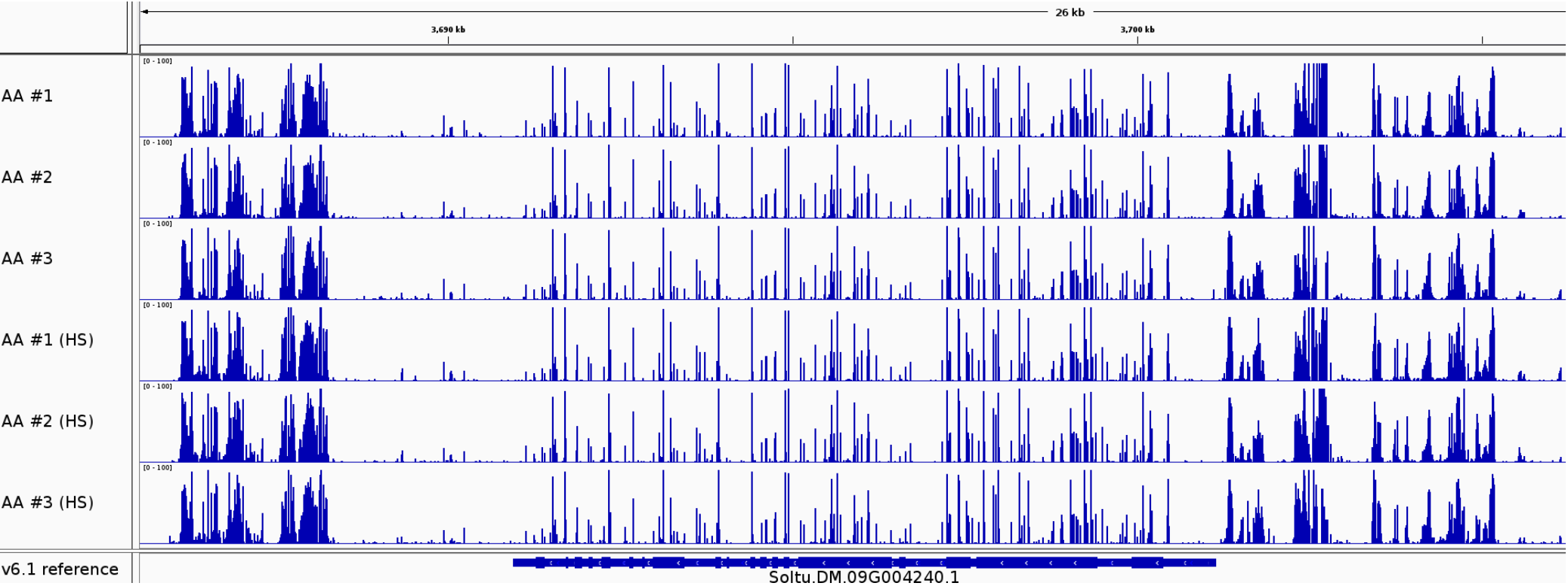

Supplement: Supplementary file 15 — Figure S15. DNA methylation levels of ROS1 gene (Soltu.DM.09G004240) within 5 kb flanking regions of Annabelle. [file TPJ-125-0-s007.pdf]

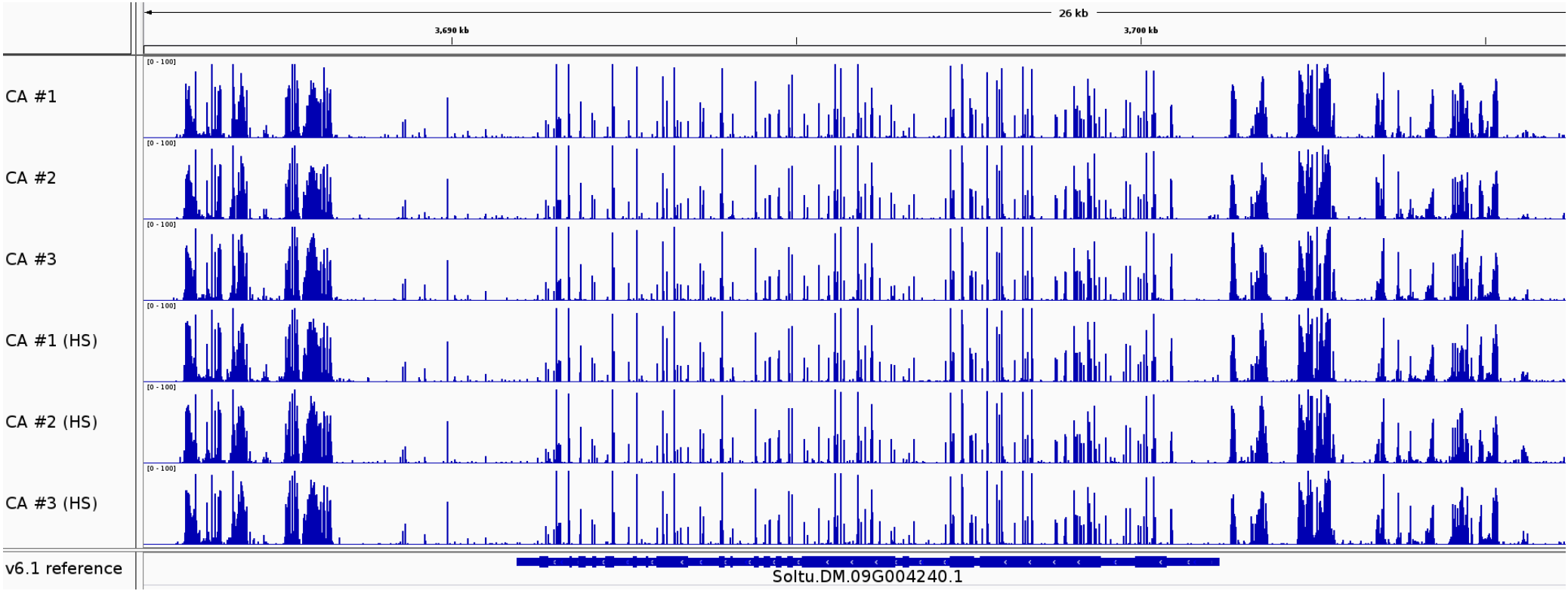

Supplement: Supplementary file 16 — Figure S16. DNA methylation levels of ROS1 gene (Soltu.DM.09G004240) within 5 kb flanking regions of Camel. [file TPJ-125-0-s003.pdf]

A

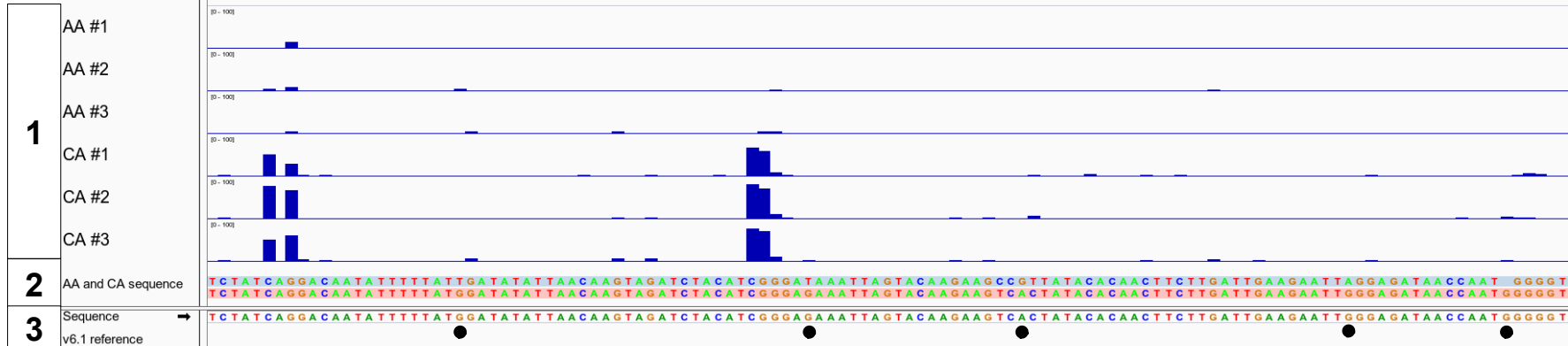

B

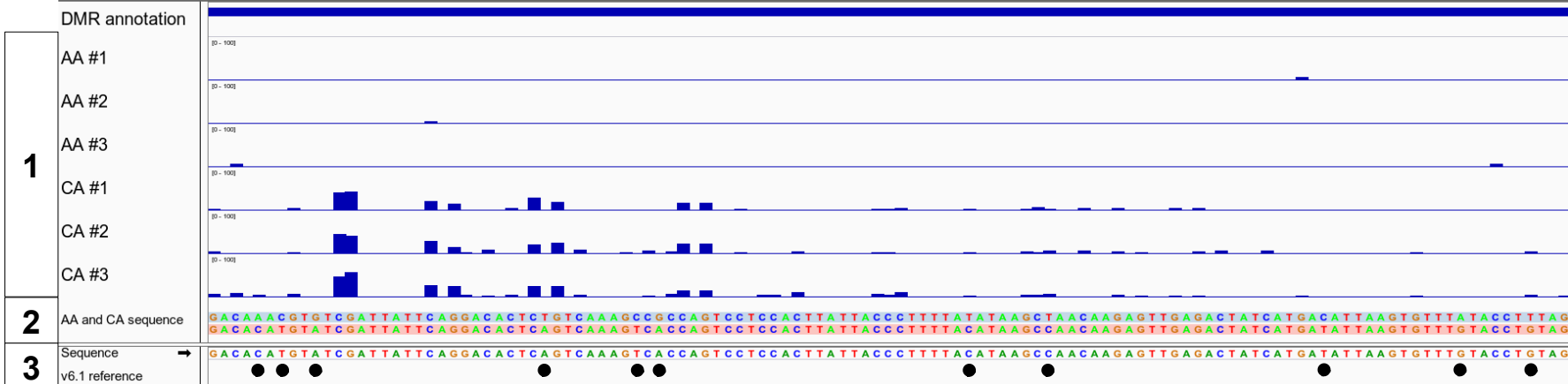

Supplement: Supplementary file 18 — Figure S18. Positions of DNA methylation sites (1) in Annabelle and Camel, the sequences (2) of Annabelle (blue bars) and Camel (red bars) and the v6.1 S. phureja reference sequence (3) within the two separate windows (A and B) of the DMR region (DMR 1) identified downstream of SP6A. [file TPJ-125-0-s006.pdf]
