# Supplementary material for: Genotype‐dependent DNA methylation patterns are negatively associated with allelic variation rather than heat‐induced gene expression in two contrasting potato genotypes
Source: Plant J. 2026 Jan 18;125(2):e70690. doi: 10.1111/tpj.70690 (PMC12812439; doi:10.1111/tpj.70690)
Supplement: Supplementary file 17 — Figure S17. Positions of DNA methylation sites (1) in Annabelle and Camel, the sequences (2) of Annabelle (blue bars) and Camel (red bars) and the v6.1 S. phureja reference sequence (3) within the DMR region (DMR 3) identified upstream of SP6A. [file TPJ-125-0-s010.pdf]

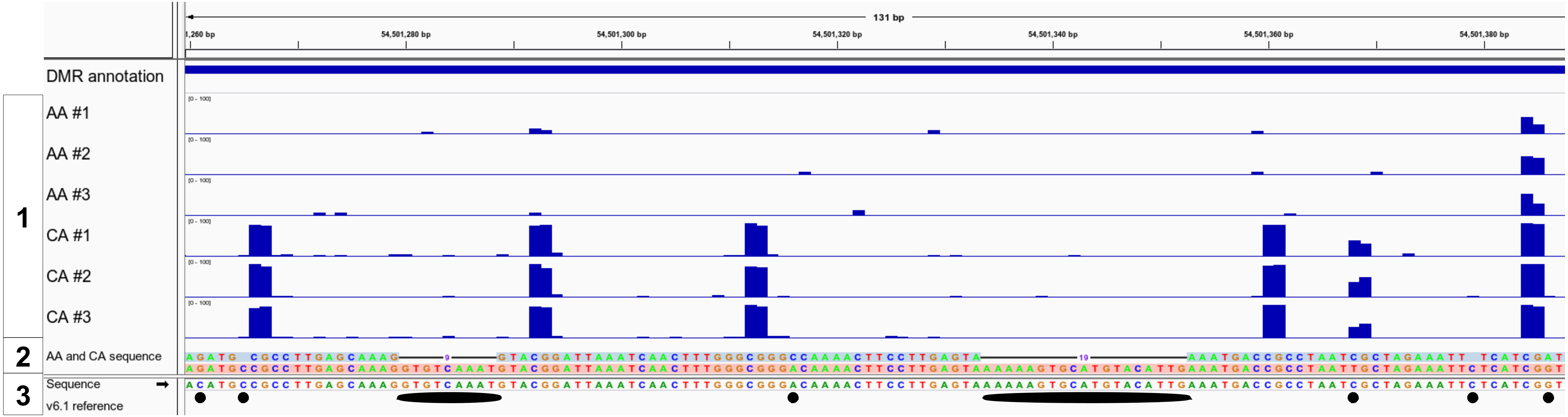

131 bp
54,501,280 bp
54,501,300 bp
54,501,320 bp
54,501,340 bp
54,501,360 bp
54,501,380 bp
DMR annotation
1
AA #1
AA #2
AA #3
CA #1
CA #2
CA #3
2
AA and CA sequence
3
Sequence
v6.1 reference
